# Supplementary material for: Hijacking of transcriptional condensates by endogenous retroviruses
Source: Nat Genet. 2022 Jul 21;54(8):1238–47. doi: 10.1038/s41588-022-01132-w (PMC9355880; doi:10.1038/s41588-022-01132-w)

nature portfolio

https://doi.org/10.1038/s41588-022-01132-w

Supplementary information

Hijacking of transcriptional condensates by endogenous retroviruses

In the format provided by the authors and unedited

SUPPLEMENTARY INFORMATION

Hijacking of transcriptional condensates by endogenous retroviruses

Vahid Asimi[1,2]†, Abhishek Sampath Kumar[1,3]†, Henri Niskanen[1]†, Christina Riemenschneider[1,3]†, Sara Hetzel[1], Julian Naderi[1], Nina Fasching[4], Niko Popitsch[4], Manyu Du[5,6], Helene Kretzmer[1], Zachary D. Smith[7,8], Raha Weigert[1], Maria Walther[1], Sainath Mamde[1], David Meierhofer[9], Lars Wittler[10], Rene Buschow[11], Bernd Timmermann[12], Ibrahim I. Cisse[5,6], Stefan L. Ameres[4,13], Alexander Meissner[1,7,8], Denes Hnisz[1*]

[*] Correspondence: hnisz@molgen.mpg.de

CONTENTS

- Supplementary Methods
- Supplementary Figure Legends
- Supplementary Table Legends
- Supplementary References
- Supplementary Figures 1-11

Supplementary Data Files 1-3 and Source Data Files 1-5 are included separately.

Supplementary Methods

Generation of the TRIM28-FKBP iPSC line

To generate mouse secondary induced pluripotent stem cells (iPSCs), secondary MEFs that harbor Doxycycline (Dox) -inducible *Oct4*, *Sox2*, *Klf4* and *c-Myc* (OSKM) transgenes, and a NANOG::GFP reporter [1], were reprogrammed. MEFs were seeded at a low density of about 10,000-25,000 cells per well of a 12-well plate that was coated with 0.2% gelatin. Prior to induction of the transgenes, cells were synchronized with a 12-16h incubation in 2.5% FBS containing medium. Afterwards, the medium was switched to 15% FBS/LIF with 2µg/ml Doxycycline. Media was changed every day; colonies emerged after about a week and clonal iPSC lines were resolved within 2 weeks. The iPSC line with the TRIM28 FKBP knock-in alleles was generated as described above.

Inactivation of NANOG::GFP in the TRIM28-FKBP iPSC line

For immunofluorescence combined with RNA FISH experiments (Fig. 3f, Extended Data Fig. 5c), GFP at the *Nanog::GFP* locus was inactivated by targeting with a guide RNA (Supplementary Table 1b) against the GFP sequence. CRISPR-Cas9-guide RNA Ribonucleoprotein complex (RNPs) was assembled following the manufacturer's guidelines (Alt-R CRISPR; IDT). Briefly, 2µl each of 200µM of crRNA and 200µM of tracrRNA was mixed and denatured at 95°C for 5 minutes, and allowed to anneal while cooling down at room temperature for 20 minutes. 3µl of crRNA-tracrRNA was mixed with 1µl of 61µM Cas9 for 20 minutes prior to transfection. TRIM28-FKBP iPSCs were transfected with the assembled RNPs by nucleofection using Amaxa 4D Nucleofector X Unit (Lonza) according to the manufacturer's instructions. The cells were then sorted for non-GFP cells using flow cytometry. The sorted cells were expanded and cultured as a clonal line.

Generation of shRNA knockdown mESC line for IAPEz and IAPEz/MMERVK10C/ /MMERVK9C/ MMETn

shRNA against the repeat elements were designed using the consensus sequence of the inner part of each element. shRNA sequences are listed in Supplementary Table 1b. shRNA sequences were cloned into an inducible plasmid following the standard cloning protocol recommended for this vector. Lentiviruses with the shRNA constructs were generated by

transfecting HEK293 cells along with packaging and envelope plasmids. TRIM28-FKBP mESCs were transduced with the virus with 8μg /mL polybrene for 48 hours followed by treatment with 2μg/ml puromycin to select for the transduced cells. Single colonies were picked, characterized and expanded as a clonal line for IAPEz and as a bulk line for the quadruple knockdown line for IAPEz, MMERVK10C, MMERVK9C, MMETn. shRNA expression was induced by treatment with 2μg/ml Doxycycline. For the experiment with pre-induction of shRNAs (Fig. 4a-d, Extended Data Fig. 6e-h), the cells were treated for 24 hours with Doxycycline followed by DMSO, dTAG-13, or Dox+dTAG-13 for another 24 hours. Tet-pLKO-puro was a gift from Dmitri Wiederschain (Novartis, Cambridge US) (Addgene plasmid # 21915). psPAX2 was a gift from Didier Trono (EPFL Lausanne) (Addgene plasmid # 12260). pCMV-VSV-G was a gift from Bob Weinberg (WIBR, Cambridge US) (Addgene plasmid # 8454). S2 work was performed following all relevant guidelines and regulations, approved by the Max Planck Institute for Molecular Genetics and the local authorities LAGeSo, Berlin (license number: 222/15-17a).

Integration of PiggyBac transposon encoding Dox-inducible ERVs

The PB-tetO-lox-GFPpA-lox-IAPEz, and -MMERVK10C constructs (Dox-inducible GFP) were created by digesting an "all-in-one" PiggyBac, TREG/Tet-3G plasmid (Addgene plasmid # 97421; a gift from Steven Carr & Samuel [Broad Institute, Cambridge US]) with restriction enzymes NcoI and KpnI and cloning the PB_ATG_GFP oligonucleotide to insert a start-codon "ATG" in front of the existing GFP via NEBuilder HiFi Assembly according to manufacturer's instructions. To integrate the loxP sites, the plasmid was digested with NheI and BamHI and integrated the sequences loxP_upstream and loxP_downstream were integrated. To integrate various IAP and MMERVK10C sequences, sequences from gDNA were amplified with primers binding in conserved regions of the ERVs and overhangs to the PiggyBac plasmid. After size selection (~900bp) fragments were cloned into the PiggyBac construct by cutting with BamHI and cloning via NEBuilder HiFi Assembly according to the manufacturer's instructions. The constructs were randomly integrated in the mESC V6.5 Trim28-FKBP subclone by co-transfecting $4x10^5$ cells with 8.5μg PB-tetO-lox-GFPpA-lox-IAPEz, or -MMERVK10C and 1.5μg Super PiggyBac transposase expression vector (SBI, PB210PA-1) using FuGENE HD Transfection Reagent (Promega). After 4 days of Puromycin selection (2μg/ml) single colonies were picked, expanded and tested for doxycycline-inducibility of the GFP construct monitored by GFP detection with FACS. Clones expressing high levels of GFP were selected and expanded for subsequent experiments. A plasmid encoding Cre recombinase

was transfected to catalyze recombination between the two homologous loxP sites, removing the GFP sequence to generate isogenic cell lines, with the same copy number and insertion sites of either GFP or IAPEz/MMERVK10C. The sequence of GFP until the polyA is about ~900bp long and comparable with the length of the IAPEz and MMERVK10C sequences.

Deletion of ERVs in the TRIM28-FKBP mESC line

To generate a TRIM28-FKBP mESC line with three ERV deletions at the *Cthrc1* locus, deletions of MMETn, MMERVK and IAP sequences were performed sequentially with CRISPR/Cas9. Guide RNAs (Supplementary Table 1b) flanking the individual ERV elements were cloned into the sgRNA-Cas9 vector pX458 and the two vectors for each cut were delivered to cells with Lipofectamine 2000 (Thermo Fisher, 11668027) according to manufacturer's instructions. Transfected cells were sorted based on GFP expression two days later with flow cytometry. Single colonies were picked, genotyped (Supplementary Fig. 6b) and clones with homozygous deletion were selected for the next ERV deletion. Deletions were also confirmed by sequencing the PCR products.

Differentiation of TRIM28-FKBP mESCs to EpiSCs (epiblast stem cells)

TRIM28-FKBP mESCs were feeder depleted and cultured for two passages under standard ESC culture conditions with 2000 U/mL LIF in 0.2% gelatin-coated 6cm dishes (Corning). 6cm dishes were coated with 10ng/mL human plasma fibronectin (Merck) and incubated overnight at 37ºC one day prior to differentiation to EpiSCs. ESCs were disaggregated for 5 minutes with Accutase (Sigma), washed twice with 1X PBS, and seeded at a density of $1x10^6$ cells per 6cm dish on fibronectin coated plates with EpiSC media containing DMEM/F12, N2B27, 1% KSR, 0.5% BSA fraction V, 1X GlutaMAX supplement, 1X non-essential amino acids, and 0.05mM ß-mercaptoethanol (R&D systems). Cells were cultured for 48–72 hours or until morphological changes were observed. At this stage, cells were passaged using a combination of TrypLE for 3 minutes and scraping with cell scrapers (Corning). EpiSCs were characterized for morphology and marker gene expression after two passages (Supplementary Fig. 2e). EpiSCs were passaged in clumps.

Differentiation of TRIM28-FKBP mESCs to iXEN (induced extra-embryonic stem cells)

TRIM28-FKBP mESCs were feeder depleted and grown on 0.2% gelatin-coated 6cm dishes for two passages under standard ESC culture conditions with 2000U/mL LIF. Cells were dissociated with 0.05 percent Trypsin (Sigma) for 4 minutes and seeded at a density of $5x10^5$

cells on a 0.2 percent gelatin coated 6cm dish with standard XEN cell media (RPMI-1640 9Invitrogen), 15% FBS (Pan biotech), 1X GlutaMAX supplement (Gibco), 1X non-essential amino acids (Gibco), 0.1mM ß-mercaptoethanol (Gibco), 0.01µM all-trans retinoic acid (sigma) and 10ng/mL Activin A (R&D systems). Cells were passaged every 3-4 days in standard XEN media until XEN-like colonies emerged. Flow cytometry was used to determine the expression of PDGFRA (1:1000, 135923, BioLegend) in iXEN cells (Supplementary Fig. 2f).

Differentiation of TRIM28-FKBP mESCs to NPCs (neural progenitor cells)

mESCs were feeder depleted and grown under standard ESC culture conditions with 2000 U/ml LIF for 5-7 days on 0.2% gelatin-coated T25 flasks (TPP). One day before neural progenitor cell (NPC) monolayer differentiation started, the cells were trypsinized using 0.05% Trypsin (Gibco, # 25300-054) for 5 min at 37°C, counted and cultured for 24 hours at high density (3-3.5 x10^6 per T25) in ESGRO Complete Clonal Grade Medium (Millipore, #SF001-B) +1000 U/ml LIF. On day 1 of differentiation cells were trypsinized as described above, washed once with PBS and plated onto 0.1% gelatin-coated 10 cm dishes (Corning) in RHB-A medium (Takara-Clontech, #Y40001) with 1.6 $\times 10^6$ cells/dish. The medium was changed on days 2 and 3 of differentiation. On day 4 of differentiation, the cells were replated on PDL/Laminin-coated 10 cm dishes at a density of 2 $\times 10^6$ cells/dish in RHB medium + bFGF (Thermo, #PHG0261). NPCs were passaged every 3 days. The dTAG treatment was performed at passage 4. Cell pellets were collected 2, 6, 24 and 96 hours post dTAG treatment initiation. DMSO-treated NPCs served as a control and were collected parallelly at corresponding time points.

Aggregation of mouse embryonic stem cells by tetraploid complementation

TRIM28-FKBP mESCs were thawed and cultured on a layer of CD-1 feeders. Prior to the complementation assay, cells were treated with either 500nM dTAG-13 or DMSO for 24 hours. Tetraploid complementation was performed as previously described [2]. For the duration of the assay, dTAG-13/DMSO was added to KSOM media. The expression of mRUBY was imaged in blastocyst stage embryos using an AxioZoom microscope (ZEISS) (Supplementary Fig. 2h-i).

Western blot

Cultured cells were lysed in RIPA buffer for 30 minutes at 4ºC, and centrifuged for 20 minutes at maximum speed. The supernatant was then transferred to a new tube and quantified by BCA

assay (Thermo Scientific). 10µg of extracted protein was run on a 4-12% NuPAGE SDS gel and transferred onto a PVDF membrane using iBlot2 Dry Gel Transfer Device (Invitrogen) according to manufacturer's instructions. The blots were blocked with 5% skim milk in TBST and incubated with primary antibodies. Primary antibodies used in this study include TRIM28 (ab22553; 1:500), ACTB (ab8226; 1:1000), OCT4 (ab19857; 1:500), OCT4 (sc-5279; 1:500), HSP90 (BD610419; 1:4000), SOX2 (ab79351; 1:500). HRP-conjugated secondary antibodies (115-035-174, 211-032-171, Jackson Immuno) were used against the host species at 1:3000 – 1:5000 dilution and visualized with HRP substrate SuperSignal West Dura (Thermo Scientific) (Fig. 1d, 3b, Extended Data Fig. 5a, 8a, Supplementary Fig 2b, 2m).

<u>Proteomics Sample Preparation and LC-MS/MS Instrument Settings for Shotgun Proteome Profiling and Data Analysis</u>

Proteomics sample preparation was done according to a published protocol with minor modifications [3]. In brief, three biological replicates of dTAG-13 -treated samples with 4 million cells per replicate were lysed under denaturing conditions and sequentially digested with LysC and Trypsin (Roche, Basel, Switzerland). Peptide desalting was performed according to the manufacturer's instructions (Pierce C18 Tips, Thermo Scientific, Waltham, MA). Desalted peptides were further separated into four fractions by strong cation exchange chromatography (SCX, 3M Purification, Meriden, CT). LC-MS/MS was carried out by nanoflow reverse phase liquid chromatography (Dionex Ultimate 3000, Thermo Scientific) coupled online to a Q-Exactive HF Orbitrap mass spectrometer (Thermo Scientific), as reported previously [4]. Briefly, the LC separation was performed using a PicoFrit analytical column (75 µm ID × 50 cm long, 15 µm Tip ID; New Objectives, Woburn, MA) in-house packed with 3-µm C18 resin (Reprosil-AQ Pur, Dr. Maisch, Ammerbuch, Germany). Raw MS data were processed with MaxQuant software (v1.6.10.43) and searched against the mouse proteome database UniProtKB with 55,471 entries, released in May 2020. The MaxQuant processed output files can be found in (Supplemental Table 3), showing peptide and protein identification, accession numbers, % sequence coverage of the protein, q-values, and label-free quantification (LFQ) intensities. The mass spectrometry data have been deposited to the ProteomeXchange Consortium (http://proteomecentral.proteomexchange.org) via the PRIDE [5] partner repository with the dataset identifier PDX021895. The correlation analysis of biological replicates and the calculation of significantly different proteins were done with Perseus (v1.6.14.0). Only groups with valid values in at least one group were used, missing values were replaced by values from the normal distribution. Statistical analysis was done by a two-sample t-test with Benjamini-

Hochberg (BH, FDR of 0.05) correction for multiple testing (Extended Data Fig. 5d, Supplementary Fig. 2c).

RNA isolation and quantitative Real-Time PCR (qRT-PCR)

RNA from cultured cells was isolated using RNeasy kit (Qiagen) according to manufacturer's instructions. 1μg of RNA was used for cDNA synthesis using RevertAid First Strand cDNA synthesis kit (Thermo Scientific) with random hexamer primers according to manufacturer's instructions. RNA from single blastocysts (E3.5) was isolated using PicoPure RNA isolation kit (Thermo Fischer Scientific) and cDNA was synthesized with High-Capacity cDNA Reverse Transcription kit (Applied Biosystems). qRT-PCR was performed with primers (Supplementary Table 1b) and 2X PowerUP SYBR green master mix (Applied Biosystems). Note that in Fig. 4a-b, and Extended Data Fig 6a-f, the qPCR primers used for MMETn and MMERVK9C elements fail to detect induction after dTAG-13 treatment, which is explained by these elements having degenerate genomic sequences.

Immunofluorescence (IF) in mouse embryos and blastocysts

E3.5 blastocysts and E6.5 embryos were dissected from the uterus in 1X HBBS and fixed in 4% Paraformaldehyde (PFA) at 4ºC overnight. The embryos were washed three times in 1X PBS with 0.1% TritonX-100 and then permeabilized in 1X SuperBlock PBS with 0.5% TritonX-100 for one hour at room temperature. Blocking against unspecific binding was done by incubating embryos in blocking buffer (1X SuperBlock PBS with 0.2% TritonX-100, 10% Donkey Serum, 3% Bovine Serum Albumin) for two hours at room temperature. Primary antibodies were diluted in antibody buffer (1X SuperBlock PBS with 0.2% TritonX-100, 10% Donkey Serum, 0.3% Bovine Serum Albumin) and incubated at 4 ºC overnight. Primary antibodies used include TRIM28 (ab22553; 1:200), OCT4 (ab19857; 1:200), NANOG (REC-RCAB002P-F; 1:400), KLF4 (AF3158; 1:200), SOX2 (ab79351; 1:200), GATA6 (AF1700; 1:200), GATA4 (sc-25310; 1:200), SOX17 (AF1924; 1:200), IAP-GAG (MBS8566075; 1:100). Embryos were washed in blocking buffer three times for 30 minutes each and incubated with host-specific secondary antibodies conjugated to fluorescent dyes for three hours in the dark at room temperature (Donkey anti-Rabbit-Alexa 488 (1:400, A21206, Invitrogen), Donkey anti-Rabbit-Alexa647 (1:400, 711-605-152, Jackson Immuno), Donkey anti-Mouse-Alexa 488 (1:400, A21202, Invitrogen), Donkey anti-Mouse-Alexa 647 (1:400, 715-605-150, Jackson Immuno), Donkey anti-Goat-Alexa 594 (1:400, A11058, Invitrogen), Donkey anti-Goat-Alexa 647 (1:400, 705-605-147, Jackson Immuno). After washing with blocking buffer,

nuclei were counterstained with 0.5μg/mL DAPI for 30 minutes in the dark at room temperature. Embryos were mounted in drops of 1X PBS and covered with mineral oil prior to imaging. Images were acquired with LSM880 (Zeiss) microscope with a 40X objective. Images were processed with and ImageJ (version 2.1.0/1.53i) (Fig. 5b, 5g Supplementary Fig. 10a-b).

SSEA-1 staining

Cultured cells were dissociated into single-cells with Accutase enzyme mix, washed in 1X PBS and fixed with 4% PFA for 15 minutes at room temperature. Following three washes with 1X PBS, the cells were incubated with fluorescent conjugated SSEA-1 antibody (BioLegend 125608, 125609; 1:1000) for one hour in the dark at 37ºC. After three washes in 1X PBS, nuclei were counterstained with 0.24 μg/ml DAPI and imaged with LSM880 confocal microscope at 63X oil objective (Supplementary Fig. 2j-k). For FACS analysis, the cells were washed two times in 1X PBS+10%FBS and analyzed with BD FACS Celesta (Supplementary Fig. 2i, 11a). Data was recorded with FACS Diva software and analyzed with FlowJo software (version 10.7)

RNA-FISH

TRIM28-FKBP iPSCs were seeded onto 0.2% gelatin-coated plates prior to treatment. For the treatment, 500nM of dTAG-13 and/or 2μg/ml Doxycycline was mixed with the media and incubated for the indicated durations. RNA-FISH was performed on glass coverslips coated with Poly-L-Lysine (Sigma-Aldrich) prior to seeding. Cells were dissociated with Accutase enzyme mix (Sigma-Aldrich), washed in 1X PBS, resuspended in a minimal volume of ESC media. 10-20μL drops of cells were added to the coverslips and were allowed to attach for 10 minutes at room temperature. 4% PFA fixation for 15 minutes was followed by two 1X PBS washes and permeabilized in 70% ethanol at 4ºC overnight. RNA-FISH was performed with reagents from the Stellaris RNA-FISH method (LGC Technologies) following the manufacturer's instructions. Hybridization was performed with an oligo probe that was labeled with Cy5 dye at the 5'end (TTCTTGATGTCCTAACCCTTTTCCTTC). After the final wash, the coverslips were mounted onto glass slides with ProLong Gold Anti-fade (Invitrogen) mounting media. Images were acquired with Z1 Observer (Zeiss) microscope at 100X magnification. Images were processed with ZEN 2.3 version 2.3.69.1016 (Zeiss) and ImageJ software version 2.1.0/1.53i (Fig. 3c-d).

Subcellular Fractionation and RNA-Isolation

A total of 1×10^6 cells was washed with ice-cold PBS, resuspended in 100 µl of Cytoplasmic Extraction Buffer (CEB) and RiboLock RNase-Inhibitor (Thermo Fisher, EO0382), incubated at 4°C for 10 min with gentle mixing. On centrifugation at 500 × g for 5 min, the supernatant (cytoplasmic extract) was transferred to a new pre-chilled tube. Pellet was resuspended in 50µl ice-cold Nuclear Extraction Buffer (NEB) and RiboLock RNase-Inhibitor (Thermo Fisher, EO0382), vortexed on the highest setting for 15 sec, incubated at 4°C for 30 min with gentle mixing, centrifuged 5000 x g for 5 min, and supernatant (nuclear extract) was transferred to a new tube for RNA extraction. CEB and NEB buffers are from the Subcellular Fractionation Kit (Thermo Fisher, 78840). For controlling the quality of RNA isolation, cDNA synthesis and PCR amplification we spiked in 10pg of a 500nt long *in vitro* transcribed RNA of *Ttn* to each fraction. RNA from cytoplasmic and nuclear fraction was isolated using miRNeasy Micro Kit (Qiagen) according to manufacturer's instructions, eluted in 25µl. 10µl of RNA from each fraction was used for cDNA synthesis using RevertAid First Strand cDNA synthesis kit (Thermo Scientific) with random hexamer primers according to manufacturer's instructions. qRT-PCR was performed with primers (Supplementary Table 1b) and 2X PowerUP SYBR green master mix (Applied Biosystems) (Extended Data Fig. 9f).

TrueSeq Stranded mRNA-seq

mESCs were cultured with either DMSO or 500 nM dTAG-13 for 2, 6, 24 and 96 hours. RNA was isolated using RNeasy kit (Qiagen) and 1µg of RNA was used for preparing the libraries. TrueSeq Stranded mRNA capture kit was used to prepare the libraries (KAPA biosystems) according to manufacturer's instructions. Unique Dual-Indexed (UDI; KAPA biosystems) adapters were ligated and the library was amplified for 8 cycles. The libraries were then sequenced as Paired-end 100 (PE100) on a Novaseq6000 with 50 million fragments per library.

Total RNA-seq

Doxycycline was used to pre-treat shRNA cell lines for 24 hours, followed by another 24 hours of treatment with DMSO, dTAG-13, or dTAG-13+Doxycycline. The RNeasy kit (Qiagen) was used to isolate RNA, and 1µg of RNA was used to prepare the libraries. Total RNAseq libraries were prepared according to the manufacturer's instructions using the KAPA RNA HyperPrep kit (KAPA biosystems). As recommended by the protocol, ribosomal RNA was depleted using the RiboErase (HMR) kit (KAPA biosystems). The library was ligated to Unique Dual-Indexed (UDI; KAPA biosystems) adapters and amplified for 9 cycles. The libraries were then

sequenced as Paired-end 100 (PE100) with a minimum of 50 million fragments per library on a Novaseq6000.

H3K27Ac and H3K9me3 Chromatin immunoprecipitation -sequencing (ChIP-Seq)

For ChIP-Seq experiments, DMSO and dTAG-13 treated cells were detached with TrypLE Express (Gibco), washed once with PBS, fixed in rotation with 1 % formaldehyde for 10 minutes in room temperature followed by 5 minutes of quenching with 125 mM glycine. For H3K27Ac and H3K9me3 ChIPs, three million mESCs were used per replicate sample, and 750,000 S2 cells were added for exogenous genome spike-in normalization [6]. Cells were lysed in LB1 (50 mM HEPES-KOH, 140 nM NaCl, 1 mM EDTA, 10 % glycerol, 0.5 % Igepal CA-630 and 0.25 % Triton X-100, 5 mM Na-butyrate and 1x protease inhibitor cocktail) and collected by centrifugation. Lysis was continued in LB2 (10 mM Tris-HCl pH 8.0, 200 mM NaCl, 1 mM EDTA, 0.5 mM EGTA, 5 mM Na-butarate and 1x protease inhibitor cocktail) followed by centrifugation. Nuclei were lysed in LB3 (10 mM Tris-HCl pH 8.0, 100 mM NaCl, 1 mM EDTA, 0.5 mM EGTA, 0.1 % Na-deoxycholate, 0.5 % N-Lauroylsarcosine, 5 mM Na-butyrate and 1x protease inhibitor cocktail) and chromatin was fragmented with Bioruptor NextGen for 35 cycles (high setting). Lysates were clarified and 10 % of the sample was set aside as input. The remaining sample was split in two to capture protein-DNA complexes with 1μg H3K27Ac (ab4729; Abcam) and 1μg H3K9me3 (ab8898; Abcam) antibodies by incubating them in rotation overnight in 4 °C. This was followed by 24 hours of incubation with Protein A Dynabeads (Invitrogen) that had been washed three times with 0.25 % BSA in PBS. Beads from immunoprecipitation were washed 7 times with RIPA buffer (50 mM HEPES-KOH pH7.5, 1 mM EDTA, 1 % Igepal CA-630, 0.7 % Na-deoxycholate, 500 mM LiCl, 5 mM Na-butyrate and 1 x protease inhibitor cocktail), once with TE buffer (10 mM Tris-HCl pH8.0, 1 mM EDTA and 50 mM NaCl) and eluted from beads with Elution buffer (50 mM Tris-HCl pH 8.0, 10 mM EDTA and 1% SDS). Samples were decrosslinked for 16 hours at 65 °C in presence of 550 mM NaCl and proteinase K (Ambion), treated with RNAse A (Thermo Scientific) and DNA was extracted with Phenol:Chloroform:Isoamylalcohol followed by chloroform extraction and ethanol precipitation. Sequencing libraries were prepared from 10 ng of DNA with KAPA HyperPrep Kit (Roche) and paired-end sequenced with NovaSeq 6000 (Illumina) to produce ~100 million fragments for each library.

RNAPII and MED23 ChIP-Seq

Cells were treated with DMSO or 500 nM dTAG-13 for 24 hours. RNAPII and MED23 ChIP-Seq samples were prepared as described above, except for MED23 samples, protein-protein crosslinking was performed by incubating cells in rotation with 2 mM disuccimidyl glutarate (Thermo Scientific; Pierce) in PBS, followed by two washes with PBS and formaldehyde fixation as above. 30 million and 10 million cells were used for RNAPII and MED23 IPs, respectively. RNAPII samples included an exogenous genome spike-in of 7.5 million S2 cells. Cell lysis was performed as above and samples were sonicated in sonication buffer (50 mM HEPES-KOH pH 7.5, 140 mM EDTA, 1 mM EDTA, 1 mM EGTA, 1 % Triton X-100, 0.1 % Na-deoxycholate, 0.1 % SDS and 1x protease inhibitors (Roche)) for 35 cycles in case of RNAPII samples and 50 cycles for MED23 samples by using Bioruptor NextGen (high setting). When preparing beads for IPs, 50 µl of Protein A Dynabeads (Invitrogen) was washed three times in blocking buffer (0.5 % BSA in PBS) and then bound to 5 µg of RNAPII antibody (8WG16; Biolegend) and 2 µg of Spike-in antibody (61686, Active Motif) or 5 µg of MED23 antibody (A300-425A; Bethyl Laboratories) during an overnight incubation in blocking buffer. Antibody-conjugated beads were washed three times with blocking buffer, resuspended to 100 µl of blocking buffer prior to adding them to sheared chromatin in a total volume of 2.5 ml sonication buffer for RNAPII and 1 ml for Med23. Immunoprecipitation was performed in rotation overnight in 4 °C. Beads were washed twice with sonication buffer, once with sonication buffer containing 500 nM NaCl, once with LiCl wash buffer (20 mM Tris pH 8.0, 1 mM EDTA, 250 mM LiCl, 0.5 % Igepal CA-630, 0.5 % Na-deoxycholate, 1x protease inhibitors) and once with TE buffer (10 mM Tris pH 8.0, 1 mM EDTA, 1x protease inhibitors). Samples were eluted from beads with 200 µl elution buffer as above and crosslinks were reversed by incubating samples in 65 °C for 16 hours. Samples were topped up with 200 µl TE buffer and 8 µl of RNAse A, incubated in 37 °C for 2 hours followed by addition of 7 µl of 300 mM $CaCl_2$, 2 µl Proteinase K (Ambion) and 30 minutes incubation in 37 °C. DNA purified from samples with phenol:chloroform:isoamyl alcohol extraction followed by ethanol precipitation. Libraries were prepared and sequenced as above for total ~50 million fragments for each library.

<u>In-situ Hi-C</u>

In-situ Hi-C experiments were performed for two biological replicates of TRIM28-FKBP mESCs after 24h DMSO or dTAG-13 treatments as described previously [7], with minor modifications. Briefly, 3 million cells per sample were fixed in suspension with 1 %

formaldehyde for 10 minutes and quenched with 0.125 M glycine for 5 minutes in rotation. Cells were washed with PBS and lysed in Hi-C lysis buffer (50 mM Tris pH 7.5, 150 mM NaCl, 5 mM EDTA, 0.5% Igepal CA-630, 1.15 % Triton X-100 and 1x protease inhibitors (Roche)) for 10 minutes on ice. Nuclei were washed with cold PBS, NEBuffer 2 (NEB) and resuspended in 50 µl of 0.5 % SDS, incubated in 62 °C for 7 minutes and SDS was quenched with 145 µl water and 25 µl 10 % Triton X-100 followed by 15 min incubation in 37 °C. For restriction enzyme digestion, 25 µl of 10X NEBuffer2 and 250 U of MboI (NEB) were added and samples were incubated overnight in 37 °C, followed by additional 1-hour incubation after adding another 250 U of MboI. Enzymes were inactivated for 20 minutes in 65 °C. Samples were spun in 5000 g, 200 µl of supernatant was removed and sample was resuspended in 200 µl of 1.2X NEBuffer 2. To fill-in DNA overhangs, 50 µl of fill-in buffer (37.5 µl of 0.4 mM biotin-14-dATP (Thermo Fisher, 19524016), 1.5 µl of dCTP, 1.5 µl of dGTP, 1.5 µl of dTTP, 10 mM each, and 8 µl of 5U/µl DNA PolI Large (Klenow) Fragment (NEB, M0210L)) was added and the samples were incubated in 37 °C for 90 minutes. For ligation reaction, samples were topped up with 663 µl of water, 120 µl of NEB T4 DNA ligase buffer (NEB), 100 µl of 10 % Triton X-100 and 12 µl of 10 mg/ml BSA (NEB, B9000S) and 2000 U of T4 DNA ligase (NEB, M0202) and incubated overnight in 18 °C in rotation. Crosslinks were reversed with Proteinase K and 0.88 % SDS in 10 mM Tris-HCl (pH 7.5) during 30 min in 55 °C, followed by incubation in 68 °C in presence of 2.3 M NaCl for 2 hours. For library preparation, DNA was extracted from samples with ethanol and sodium acetate precipitation, sheared with Covaris E220 and pulled down with 150 µl of T1 Streptavidin Dynabeads (Thermo Fisher). The end-repair for captured DNA was performed at room temperature for 30 minutes using 100 µl reaction with 0.5 U of T4 Polynucleotide Kinase (NEB), 12 U of T4 DNA polymerase (NEB) 5 U of DNA pol I Large (Klenow) Fragment in NEB T4 DNA ligase buffer with 10 mM ATP and 0.5 mM dNTP. After washing the bead-bound DNA, samples were resuspended to NEBuffer 2 with 0.5 mM dATP and 25 U of Klenow exo minus (NEB, M0212) and incubated in 37 °C for 30 minutes. After washing the beads, universal sequencing adapters from NEBnext Ultra DNA Library Kit (NEB) were ligated in NEB Quick ligation reaction buffer according to manufacturer's guidelines. Libraries were amplified for 6 PCR cycles and purified using Ampure XP beads (Beckman Coulter). Libraries were paired-end sequenced with NovaSeq 6000 for 400 million fragments for each library.

Generation of DNA constructs for protein purification

For the purification of fluorescently labeled fusion proteins we amplified the C-terminal domain of POLR2A, full-length CBX5 (HP1α), the intrinsically disordered region of MED1 and the intrinsically disordered region of NFYC from cDNA generated from v6.5 mouse embryonic stem cells. The amplified fragments were cloned into pET45-mCherry (Addgene Cat# 145279) or pET45-mEGFP (this paper) backbones by Gibson assembly using NEBuilder® HiFi DNA Assembly Master Mix (NEB). All sequences of interest were cloned C-terminally to the respective fluorescence marker.

Protein purification

Protein overexpression in BL21 (DE3) (NEB M0491S) was performed as described [8]. Pellets were resuspended in 25 mL of ice-cold Buffer A (50 mM Tris pH 7.5, 500 mM NaCl, 20 mM Imidazole) supplemented with cOmplete protease inhibitors (Sigma, 11697498001) and sonicated for 10 cycles (15 s ON, 45 s OFF) on a Qsonica Q700 sonicator. The resulting lysate was cleared by centrifugation at 15,500 g for 30 minutes at 4°C. For protein purification we used the Äkta Avant 25 chromatography system. The supernatant was loaded onto a cOmplete His-Tag purification column (Merck, 6781543001) pre-equilibrated in Buffer A. The loaded column was washed with 15 column volumes (CV) of Buffer A. Bound fusion protein was eluted in 10 CV of Elution Buffer (50 mM Tris pH 7.5, 500 mM NaCl, 250 mM Imidazole) and immediately diluted 1:1 in Storage Buffer (50 mM Tris pH 7.5, 125 mM NaCl, 1 mM DTT, 10% Glycerol). The resulting eluate was concentrated by centrifugation at 10,000 g for 30 minutes at 4°C using 3000 MWCO Amicon Ultra centrifugal filters (Merck, UFC803024). The concentrated fraction was diluted 1:100 in Storage Buffer, re-concentrated and stored at -80°C.

In vitro transcription of IAP RNA, seRNA, MSR RNA

To generate *in vitro* transcribed RNA for the *gag* region of IAPEz, template PCR amplicon was generated from cDNA using primers listed in Supplementary Table 1b. The regions of interest cloned for in vitro transcription of the *miR290-295* super-enhancer RNA and Major Satellite Repeat RNA were previously described [9,10], and the primers used are listed in Supplementary Table 1b. 1μg of DNA was used for *in vitro* transcription using Fluorescein 12-UTP or Cy5-UTP RNA labeling kit (Jena Biosciences) and incubated at 37ºC for 4 hours followed by 15mins incubation with TURBO DNaseI. Labeled RNA was purified using Clean and Concentrator Kit-5 (Zymo research) and eluted in Tris-HCl pH8.0. For *mir290-295* super-enhancer RNA, transcribed RNA of positive and negative strand was mixed 1:1 at equimolar

concentrations. The size IAPez GAG RNA: 820-880 bp, Major Satellite Repeat: 450-550 bp, *miR290-295* seRNA: 930 bp.

In vitro droplet assay

For the *in vitro* droplet formation assays (Fig. 4e-j, Extended Data Fig. 7, 8), the purified mCherry- or mEGFP-fusion proteins were measured for concentration and subsequently diluted to the desired concentration in Storage Buffer (50 mM Tris pH 7.5, 125 mM NaCl, 1 mM DTT, 10% Glycerol). Protein solutions were mixed with Fluorescein- or Cy5-labeled RNA and then diluted 1:1 with 20% PEG-8000 in de-ionized water (w/v). After 30 minutes of incubation at room temperature, 10 µl of this mix was pipetted onto a chambered coverslip (Ibidi, 80826-90). Images were acquired using an LSM880 confocal microscope equipped with a Plan-Apochromat-63x/1.40 oil DIC objective with a 2.5x post-magnification if indicated. Data for the quantification of condensate formation in *in vitro* mixing assays was acquired from at least 5 images of two independent image series per condition. For the detection of droplet regions, we used the ZEN blue 3.1 Image Analysis and Intellesis software packages. Image segmentation was achieved by use of a previously trained ZEN Intellesis algorithm for classification of each individual pixel into foreground (droplet area) or background (image background). Generated probability maps with a minimal confidence of at least 90%, a minimum area of 3 pixels and watershed for primary objects were implemented into the ZEN Image Analysis module to classify regions of interest. For the calculation of the partition ratio, the mean intensity values of droplets and background for the respective channel was used. Enrichment of RNA in droplets was calculated by background subtraction from the mean intensity values of droplet areas in the respective RNA channel (Fig. 4g, 4j, Extended Data Fig. 7e) or as partition ratio as described above. All figures were generated using R-Studio.

RNA-Seq processing

Raw reads were subjected to adapter and quality trimming with cutadapt [11] (version 2.4; parameters: --nextseq-trim 20 --overlap 5 --minimum-length 25 --adapter AGATCGGAAGAGC -A AGATCGGAAGAGC), followed by poly-A trimming with cutadapt (parameters: --overlap 20 --minimum-length 25 --adapter "A[100]" --adapter "T[100]"). Reads were aligned to the mouse reference (mm10) using STAR [12] (version 2.7.5a; parameters: --runMode alignReads --chimSegmentMin 20 --outSAMstrandField intronMotif --quantMode GeneCounts) and transcripts were assembled using StringTie [13] (version 2.0.6; parameters: -e)

with GENCODE annotation (VM19). For the repeat expression quantification, reads were re-aligned with additional parameters '--outFilterMultimapNmax 50'.

Public ChIP-Seq data

Fastq files of public ChIP-Seq data for H3K27Ac [14], H3K9me3 [15], HP1α [16], TRIM28 [15], NANOG [17], OCT4 [17] and SOX2 [17] were downloaded from GEO (Supplementary Table 1c).

ChIP-Seq processing

Raw reads of treatment and input samples were subjected to adapter and quality trimming with cutadapt (version 2.4; parameters: --nextseq-trim 20 --overlap 5 --minimum-length 25 --adapter AGATCGGAAGAGC -A AGATCGGAAGAGC). Reads were aligned separately to the mouse genome (mm10) and to the fly genome (*D. Melanogaster*, dm6) using BWA [18] with the 'mem' command (version 0.7.17, default parameters). A sorted BAM file was obtained and indexed using samtools with the 'sort' and 'index' commands (version 1.10). Duplicate reads were identified and removed using GATK [19] (version 4.1.4.1) with the 'MarkDuplicates' command and default parameters. Technical replicates of treatment and input samples were merged respectively using samtools 'merge'. Peaks were called with reads aligning to the mouse genome only using MACS2 [20] 'callpeak' (version 2.1.2; parameters --bdg --SPMR) using the input samples as control samples. For H3K9me3 only, the '--broad' option was used. Genome-wide coverage tracks for single and merged replicates normalized by library size were computed using deepTools bamCoverage (parameters: --normalizeUsing CPM --extendReads) and in addition normalized by the spike-in factor obtained from the reads aligning to the *Drosophila* genome as described [6].

Publicly available paired-end ChIP-Seq data (HP1α) were processed as described above except spike-in and input normalization.

Publicly available single-end ChIP-Seq data (H3K9me3, TRIM28, H3K27ac, NANOG, OCT4, SOX2) were trimmed using cutadapt (version 2.4; parameters: --nextseq-trim 20 --overlap 5 --minimum-length 25 --adapter AGATCGGAAGAGC), and aligned and de-duplicated analogous to the paired-end data. In order to estimate the fragment size, only reads with a mapping quality of at least 15 were considered and used as input for spp [21] (version 1.2.2). All reads were then used to create coverage tracks using bamCoverage and to call

peaks with MACS2 ‘callpeak’ (version 2.1.2; parameters --bdg --SPMR --nomodel) with ‘--extsize’ set to the estimated fragment length divided by two and input samples used as control for H3K27ac, NANOG, OCT4 and SOX2. For H3K9me3 only, the ‘--broad’ and ‘--nolambda’ options were used.

The co-ordinates displayed for ChIP-Seq tracks in figures all correspond to mm10 genome assembly co-ordinates.

<u>Detection of eRNA expression and preparation of TT-SLAM-Seq histograms</u>

TT-SLAM-Seq signal was quantified at typical enhancer and SE constituents. Enhancers within 3kb of TSS were excluded and intragenic enhancers were only considered on opposing strand relative to genes (GENCODE VM19, excluding “bidirectional_promoter_lncRNA”) to measure eRNA without interference from gene transcription. Gene coordinates were extended for 10kb from transcription termination site to prevent overlap of transcriptional readthrough signal. The detection of eRNA was performed at 2 kb regions centered by the constituent coordinates. TT-SLAM-Seq counts were quantified from .bam files with htseq-count (parameters: --stranded = yes, --nonunique = all). Differential expression analysis was performed on enhancer elements with DEseq2 [22] (parameters: test="LRT", reduced=~1) and excluding regions with less than 10 counts across samples. For visualizing fold changes, regions with low eRNA expression (FPKM < 1) were excluded (Fig. 1i). In Fig. 1i, enhancer constituents with significant transcription (FPKM > 1) are considered (n = 117 for super-enhancers, n = 153 for typical enhancers).

Quantifications for eRNA histograms were done with HOMER software [23] (version 4.10) and only intergenic enhancers were considered. Reads from .bam files were prepared for quantifications with ‘makeTagDirectory’, three replicates were merged, and histograms for enhancer and SE constituents were prepared with ‘annotatePeaks.pl’ (parameters: -size 4000, -hist 50) and metagene plots for SEs with ‘makeMetaGeneProfile.pl’ (parameters: -min 500, -size 4000). Histograms were smoothened by taking a rolling mean with window size 5 for enhancer constituents and 3 for SEs before plotting. To visualize TT-SLAM-Seq signal across genes, protein-coding genes were considered and metagene plot was prepared using ‘makeMetaGeneProfile.pl’ after removing outliers (FPKM <0.5 or >100). Histograms were

smoothened by taking a rolling mean with window size 3 before plotting (Supplementary Fig. 3c).

To compare relative levels of eRNAs and transcripts derived from repeat elements (Extended Data Fig. 1d), reads were prepared for quantifications using ‘makeTagDirectory’ with -keepOne option, allowing multimapping reads to be considered only once. Reads at intergenic retrotransposons belonging to LTR class (Repbase [24]) were quantified using ‘analyzeRepeats.pl’ and LTRs with less than 5 reads across 0h and 24h samples were discarded. To analyze putative upregulated LTRs (“UP LTRs”), differential expression was estimated using DEseq2 (parameters: test="LRT", reduced=~1). LTRs with fold change above 2 and adjusted p-value < 0.05 were considered. To visualize TT-SLAM-Seq signal, coverage tracks from both strands were prepared from HOMER TagDirectories with ‘makeUCSCfile’, converted to bigwig format using ‘bedGraphToBigWig’ and metaprofile plots were prepared using EnrichedHeatmap [25] as described for ChIP-Seq below.

To quantify eRNA expression from total RNA-Seq data (Fig. 4c), aligned reads were prepared for quantification with HOMER ‘makeTagDirectory’ and quantified with 'analyzeRepeats.pl' using the enhancer constituents defined above. Mean FPKM values from three replicates were calculated for each enhancer and regions with low read counts (FPKM < 0.05) were excluded from analysis.

Differential gene expression analysis

For the differential gene expression analysis only protein coding genes were considered. Differential expression for RNAseq and TT-SLAM-Seq samples was measured using DESeq2 (parameters: test="LRT", reduced=~1) based on the raw counts per gene considering all time points per experiment type in one design. Only genes with at least 10 reads across all samples of the same experiment type were considered for the analysis. Genes with an absolute log2 fold change greater than 1 in comparison to the DMSO control and an adjusted p-value of less than 0.05 were termed differentially expressed per time point. Lowly expressed genes across all time points (average TPM across all RNAseq samples of less than 0.5 or average FPKM across all TT-SLAM-Seq samples of less than 0.25) were excluded from the analysis.

Gene set enrichment (GSEA) analysis

GSEA was performed to test if super-enhancer (SE) associated genes were overrepresented in the sets of TT-SLAM-Seq differentially regulated genes. All protein-coding genes were ranked based on log2 fold change. To prepare a gene set for the GSEA, Refseq IDs of SE-associated genes obtained from [26] were converted to (version free) Ensembl gene IDs using biomaRt R package [27]. GSEA was run with the fgsea R package [28] using 100000 permutations (Fig. 1h).

Mappability

The genome-wide mappability was calculated using GenMap [29] (parameters: -K 100 -E 0 -t -w -bg) with the mouse genome (mm10) as input (Supplementary Fig. 1c-g).

ChIP-Seq enrichment analysis

Bigwig tracks of ChIP-Seq signal were imported into R with the package rtracklayer [30]. Enriched heatmaps and metaprofile plots of ChIP-Seq signal were generated using the R package EnrichedHeatmap [25]. For this purpose, the signal was normalized to genomic features using the function ‘normalizeToMatrix’ (parameters: extend = c(2000, 2000), mean_mode = “w0”, w = 50, target_ratio = 0.25). The resulting data matrix was visualized using the function ‘EnrichedHeatmap’.

Hi-C data processing and analysis

Raw reads were trimmed for adapters and quality as described above. Reads were further trimmed at MboI restriction site using cutadapt (version 2.4; parameters: -a GATC, --minimum-length 25) and separately aligned to the mouse genome (mm10) using BWA [18] ‘mem’ (version 0.7.17, default parameters). Replicates were processed individually and then merged with samtools ‘merge’ [31]. Remainder of Hi-C data processing was performed with HOMER software [23] (version 4.10), unless specified otherwise. Paired-end reads were matched and prepared for analysis with ‘makeTagDirectory’ and PCR duplicates were removed (parameters: -tbp 1). Reads were filtered to remove continuous genomic fragments, re-ligation events, self-ligations and tags from regions with unusually high tag density while keeping only paired-end reads where both reads have a MboI restriction site within the fragment length estimate 3’ to the read (parameters: -removePEbg -restrictionSite GATC -both -removeSelfLigation -removeSpikes 10000 5).

Analysis of compartments was performed with HOMER ‘runHiCpca.pl’ using resolution of 25 kb, 50 kb analysis window and using H3K27Ac peaks to assign active (A) and inactive (B)

compartments. Hi-C matrices were prepared for visualization with 'analyzeHiC' by normalizing counts to interactions per hundred square kilobases per billion (default) or displaying log2 observed/expected counts normalized for interaction distance (parameters: -distNorm). Resolutions used were 300 kb for whole chromosome view (Supplementary Fig. 6a), 150kb resolution and 300 kb 'window' for region chr12:3,600,000-35,800,000 (Supplementary Fig. 6b, left) and in displaying differential (dTAG-13 - DMSO) of observed/expected matrix (Supplementary Fig. 6b, right), 10 kb resolution for region chr15:38,300,000-39,800,000 (Supplementary Fig. 6c), 25 kb resolution for region chr7: 26,037,000-31,262,000 in (Supplementary Fig. 8a) and 5kb resolution and 20kb 'window' for region chr7:29,260,000-30,000,000 in (Supplementary Fig. 8b). Images were prepared with Treeview 3.0

To perform pile-up analysis from ERV-gene and ERV-super-enhancer contacts, HOMER formatted Hi-C data was converted first to .hic format with 'tagDir2hicFile.pl' and then to .cool format with 'hic2cool convert' (version 0.8.3). Pile-up analysis was performed with 'coolpup.py' (version 0.9.5) software [32] using Knight-Ruiz balanced matrices with 50 kb resolution and analyzing all cis interactions between ERVs and super-enhancers and between ERVs and protein coding genes, excluding genes with low expression (FPKM < 0.25 across TT-SLAM-Seq samples).

Motif enrichment

Enrichment of motifs was calculated using ame (version 5.3.0, default parameters) [33]. The sequences of 5' full length IAP LTRs and full length IAP inner parts were analyzed separately using the sequences of super-enhancer constituents as control (Extended Data Fig. 4b). In Extended Data Fig. 4b, the top TFs whose motifs show enrichment in the IAP LTRs or inner parts are shown. Also displayed is the expression level of the TFs calculated from the RNA-Seq data. For further functional tests, NFY was selected as its motif is highly enriched in IAPs, and it is expressed above 50 TPM in mESCs.

Genotyping

Genotyping was performed as described [34]. Briefly, samples were aligned against a hybrid mouse assembly (mm10 with positions of SNPs with the CAST/Eij strain masked as 'N' using SNPsplit [35] using STAR (parameters: --outSAMattributes NH HI NM MD). Reads were sorted by reference genome using SNPsplit. SNPs identified in [34] to be covered by reads from both

genomic backgrounds were used to determine the allele composition for each cell. Individual cells were then assigned to embryos using the autosomal distribution of CAST/Eij SNPs. In order to determine embryo sex, marker genes *Xist*, *Erdr1*, *Ddx3y* and *Eif2s3y* were used to evaluate X and Y chromosome transcription based on the counts produced by Cell Ranger. Embryos were determined to be female if they had a high fraction of cells expressing *Xist*, while embryos with a high faction of cells expressing *Erdr1*, *Ddx3y* and *Eif2s3y* were determined to be male. Cells that could not confidently be assigned to an embryo were discarded from the analysis.

Wild-type reference

In order to define clusters of cells for the wild-type embryos, the R package Seurat (v3.2.3) [36] was used. For each time point (E5.5, E6.5 and E7.0), the 3000 most variable genes were detected across all cells and embryos. All time points were integrated using the functions 'FindIntegrationAnchors' and 'IntegrateData' (parameters: dims = 1:20). The combined wild-type time points were log2-normalized ('NormalizeData') and scaled while accounting for potential bias from cell cycle or mitochondrial gene counts using the function 'ScaleData' with parameters 'vars.to.regress = c('percent.mt', 'S.Score', 'G2M.Score')'. A UMAP was used to represent the cells in two dimensions using the function 'RunUMAP' (parameters: reduction = 'pca', dims = 1:20) based on the PCA computed by the function 'RunPCA' (parameters: npcs = 30). Six clusters of cells were identified using the functions 'FindNeighbors' (parameters: reduction = 'pca', dims = 1:20) and 'FindClusters' (parameters: resolution = 0.1). Marker genes per cluster were identified with the 'FindAllMarkers' function (parameters: only.pos = TRUE, min.pct = 0.25, logfc.threshold = 0.25). Based on the detected marker genes per cluster and literature-based markers of cell states we assigned clusters to the following cell states: epiblast, primitive streak, extraembryonic ectoderm (ExE)/trophoblast, visceral endoderm, mesoderm and parietal endoderm (Supplementary Table 6). The heatmap of marker genes per cell state was generated based on the z-score transformed log2-normalized counts using the function 'DoHeatmap' (Extended Data Fig. 10f).

Knockout assignment

The E6.5 TRIM28 knockout expression data were log2-normalized analogous to the wild-type time points and transferred to the combined wild-type reference using the functions 'FindTransferAnchors' (parameters: dims = 1:30) and 'TransferData' (parameters: dims = 1:30) based on the cell states identified in the wild-type. Wild-type and knockout samples were

integrated using ‘FindIntegrationAnchors’ and ‘IntegrateData’ (parameters: dims = 1:20, k.filter = 50). Analogous to the wild-type reference, the combined dataset was scaled and a UMAP was used in order to represent the data in two dimensions (same parameters for all functions as for the wild-type reference).

Cell state proportions

Per embryo, the proportion of a cell state was computed as the number of cells assigned to a cell state divided by the total number of cells assigned to the respective embryo. Per time point, cell state proportions were represented by the median of proportions of the respective cell state across all embryos of a developmental time point (Fig. 5d, Supplementary Fig. 9a-b).

RNA velocity

RNA velocity was computed using velocyto (v0.1.18) [37], visualized using scanpy (v1.4.3) [38], and projected on the wild-type reference UMAP (Extended Data Fig. 10e).

Cut site analysis

Cut site analysis of the guide RNA target regions was performed using the sequence reads from the E6.5 wild type and E6.5 TRIM28 KO embryos, as described previously [34]. The aligned reads were then classified according to their alignment to the sgRNA target site as follows: (1) 'spliced/deleted' if they did not match any nucleotide but spanned the entire target site, (2) 'mismatched' if any of the nucleotides were aligned as a mismatch/deletion/insertion to the reference, (3) 'complete' if all nucleotides matched the target site (Extended Data Fig. 10b).

Pluripotency and differentiation marker quantification

For the pluripotency and differentiation marker genes, TPMs were z-score transformed and visualized using the R package and function pheatmap [Kolde (2019) pheatmap: Pretty Heatmaps. R package version 1.0.12. https://CRAN.R-project.org/package=pheatmap] (Supplementary Fig. 11b-c).

Supplementary Figure Legends

Supplementary Fig. 1. TRIM28 occupies ERVs but not enhancers and promoters in mESCs

a. Classes of retrotransposons in the mouse

b. (top) Model of TRIM28/HP1α pathway and its major regulators at endogenous retroviruses (ERVs). KRAB-ZFP: KRAB-Zinc Finger Protein, TRIM28: Tripartite motif - containing protein 28, HP1α: heterochromatin protein 1α. (bottom) Model of enhancers. Enhancers are bound by transcription factors (TFs) that recruit the Mediator complex and RNA Polymerase II (RNAPII).

c-j. Heatmap representation of ChIP-seq read densities of the indicated factors within a 2kb window around the indicated genomic features. The genomic features (the middle part of the plot) were length normalized. Meta-analyses of the mean binding profile of the indicated factors are displayed above the heatmaps. Enhancers in mESCs are bound by the OCT4, SOX2, NANOG TFs, and are devoid of components of the TRIM28/HP1α pathway that are bound at ERVs. For transposable elements, the mappability information is displayed on the right. Rpm: reads per million.

Supplementary Fig. 2. Extended characterization of TRIM28-FKBP ESCs

a. Scheme of FKBP knock-in strategy at the *Trim28* locus.

b. Western blot validation of the FKBP degron tag and its ability to degrade TRIM28. Actin is shown as the loading control.

c. Mass spectrometry analysis of protein levels after 24h of dTAG-13 treatment. Every dot corresponds to a quantified protein. The degradation appears highly selective for TRIM28.

d. Scheme of directed differentiation to Epiblast stem cells (EpiSCs), induced Extra-embryonic stem cells (iXEN) and Neural Progenitor cells (NPCs). Differentiation experiments were performed with TRIM28-FKBP mESC clone 1.

e. EpiSCs can be derived from TRIM28-FKBP ESCs. Representative bright field images demonstrating the morphology of undifferentiated mESCs and derived EpiSCs. Differentiation was performed three times independently. Images displayed from one representative experiment Scale bar is 200μm.

f. iXEN can be derived from TRIM28-FKBP ESCs. Flow cytometry analysis of PDGFRA expression in undifferentiated mESCs and derived iXEN cells. The dotted line indicates the gating strategy used to categorize the population as negative or positive. The barplot depicts the percentage of PDGFRA positive cells that correspond to each condition. The experiment was performed in triplicates, and a representative quantification is shown.

g. TRIM28-FKBP ESCs can be differentiated to NPCs. Marker gene expression in parental line, TRIM28-FKBP mESC, and NPCs derived from TRIM28-FKBP mESC. Heatmap representation of RNA-seq read density (TPM) $\log_2$ fold change (across 3 replicates for each) of pluripotency and neural marker gene expression.

h. Scheme of tetraploid complementation assay. TRIM28-FKBP mESC was treated with dTAG-13 for 24 hours prior to aggregation. dTAG-13/DMSO was added to the aggregation media.

i. TRIM28-FKBP ESCs contribute to the ICM (inner cell mass)/pluripotent lineage, which is not observed in prolonged presence of dTAG-13. In blastocyst stage embryos, morphology and mRUBY expression were determined. The expression of mRUBY identifies TRIM28-FKBP ESCs. The dotted line represents the ICM (inner cell mass), the embryo's pluripotent lineage. Experiment was performed in triplicates and the ‘n/n’ denotes the number of embryos that showed mRUBY staining / the number of embryos profiled. Scale bar is 20μm.

j. Scheme of the SSEA-1 staining experiments. SSEA-1 is a marker of pluripotent cells. Withdrawal of LIF in the cell culture medium leads to differentiation, and is included as a control.

k. 24h of dTAG-13 treatment does not substantially change the fraction of cells expressing the SSEA-1 pluripotency marker. Displayed are representative images of SSEA-1-Cy5 from one immunofluorescence experiment. Scale bars: 50µm.

l. FKBP allele knock-in has no effect on the pluripotent state of ESCs. Flow cytometry analysis of the respective pluripotency marker SSEA-1-stained cell lines. The dashed line denotes the gating for low and high SSEA-1 expression, respectively. The percentage of cells expressing high levels of SSEA-1 is indicated by the numbers.

m. Western blot validation of the levels of the pluripotency TF OCT4 in two independent TRIM28-FKBP mESC clones.

n. TRIM28-FKBP ESCs have a similar transcriptional profile to parental V6.5 cells. The heatmap demonstrates a strong correlation between parental and knock-in ESCs (Pearson correlation). Correlation coefficients were calculated between ESCs and NPCs using a set of 6,736 differentially expressed genes.

Supplementary Fig. 3. TT-SLAM-Seq detects nascent transcription

a. Schematic overview of the TT-SLAM-Seq experimental and analytical procedure.

b. TT-SLAM-Seq and H3K27Ac ChIP-Seq browser tracks at the *Klf4* super-enhancer locus. Rpm: reads per million. Co-ordinates are mm10 genome assembly co-ordinates.

c. TT-SLAM-Seq detects nascent transcription at genes, enhancers and super-enhancers. Displayed are meta representations of TT-SLAM-Seq read densities from + and – strands at genes, super-enhancers, constituent enhancers of super-enhancers, and enhancers in mESCs. The genomic features at genes and super-enhancers were length normalized. Reads are normalized to 10^7 mapped reads and displayed as tags per bp per peak.

Supplementary Fig. 4. Retrotransposon de-repression in TRIM28-degraded ESCs

a. Classes of retrotransposons in the mouse.

b. Transcript levels of ERVs, LINEs and SINEs detected with TT-SLAM-Seq and RNA-Seq upon dTAG-13 treatment in mESCs. Values are normalized to the levels detected at 0h. Data are from three biological replicates.

c. Transcript levels of ERV (sub)classes detected with TT-SLAM-Seq and RNA-Seq upon dTAG-13 treatment in mESCs. Values are normalized to the levels detected at 0h. Data are from three biological replicates.

d. Transcript levels of IAP subfamilies detected with TT-SLAM-Seq and RNA-Seq upon dTAG-13 treatment in mESCs. Values are normalized to the levels detected at 0h. Data are from three biological replicates.

e. Transcript levels of the indicated ERV taxa detected with TT-SLAM-Seq and RNA-Seq upon dTAG-13 treatment in mESCs. Values are normalized to the levels detected at 0h. Data are from three biological replicates.

f. qRT-PCR analysis of *IAPEz* RNA levels upon dTAG-13 treatment in mESCs. Values are normalized to the levels detected at 0h. Data are from three independent biological replicates (i.e. three wells on a tissue culture plate), and are presented as mean values +/- SD. The experiment was repeated three times. P-values are from two-tailed t-tests. *:P=0.023, ***:$P<10^{-3}$.

g. Reduced H3K9me3 at IAPs, MMERVKs and MMETns in dTAG-13 treated (24h) mESCs. Displayed are heatmap representations of H3K9me3 ChIP-seq read densities within a 2kb window around the indicated genomic features. The genomic features (the middle part of the plot) were length normalized. Meta-analyses of the mean binding profile of the indicated factors are displayed above the heatmaps. Rpm: reads per million.

Supplementary Fig. 5. Reduced occupancy of transcription-associated factors at super-enhancers and increased occupancy at ERVs in TRIM28-degraded mESCs

a. Genome browser tracks of ChIP-Seq data (H3K27Ac, RNAPII, MED23) in control mESCs and upon 24h dTAG-13 treatment at the *miR290-295* locus. Rpm: reads per million.

b. Heatmap and meta representations of H3K27Ac, MED23 and RNAPII ChIP-Seq read densities at IAP, MMERVK and MMETn ERVs in control (DMSO) and dTAG-13 (24h) treated mESCs. The mean read densities are displayed +/-2kb around the indicated elements. The genomic elements (the middle part of the meta plot) were length normalized.

Supplementary Fig. 6. Characterization of gene expression at the *Cthrc1* locus after CRISPR/Cas9-mediated deletion of three ERVs

a. The *Cthrc1* locus, highlighting the three ERVs that are deleted in the ERV TKO mESC line. TRIM28 ChIP-Seq data is shown above the gene models.

b. Genotyping PCR of the ERV TKO cell line. Genotyping was performed once.

c. Chromatin contacts at the *Cthrc1* locus. Displayed is the interaction matrix from in-situ Hi-C experiment from DMSO control samples. Positions of gene coordinates are displayed below the matrix and genes analyzed in **d-e** are annotated. A thick white stripe in the interaction matrix represents an error in mm10 genome assembly.

d. RT-qPCR analysis showing relative expression of *Cthrc1*-proximal genes in ERV TKO cell line from six biological replicates. Bar charts depict the mean and error bars standard deviation.

e. RT-qPCR analysis of gene expression of *Cthrc1* and proximal genes in response to 24h dTAG-13 treatment from six biological replicates. *IAPEz* and *pri-MiR290-295* are shown as controls. Values are normalized to the DMSO control. Bar charts depict the mean and error bars standard deviation. P-values are from two-tailed t-tests.

Supplementary Fig. 7. TRIM28-degradation leads to shift of ERVs from the inactive "B" towards the active "A" compartment

a. TRIM28-degradation does not lead to major genome-wide changes in chromatin contacts. Displayed are the Hi-C interaction matrices for chromosome 12, and the Principal Component 1 (PC1) of the normalized interaction matrix of the DMSO control and dTAG-treated samples.

b. (left) Hi-C interaction matrices for chromosome 12, zoomed-in on an ERV cluster, and the Principal Component 1 (PC1) of the normalized interaction matrix of the DMSO control and dTAG-treated samples. (right) differential interaction matrix of the region shown on the left.

c. TRIM28-degradation leads to a shift of ERVs from the inactive "B" towards the active "A" compartment. Quantification of the change in mean compartment score of the indicated genomic regions. UP-LTRs are the putative upregulated ERVs displayed in Extended Data Fig. 1d, UP and DOWN genes are the differentially regulated genes from Fig. 1g (24h), non-DE genes are active genes not differentially expressed. One thousand largest H3K9me3 peaks were considered as an additional control. The values are displayed as the difference between the PC1 value in the dTAG-treated sample and the DMSO control sample. The number of elements in each category in shown in parentheses.

d. Pile-up analysis of contacts between IAPs, MMERVKs, MMETns, UP-LTRs (see above), and (top) transcribed genes or (bottom) super-enhancers (SEs) in wild type and TRIM28-degraded mESCs. The "all ERVs set" includes the combination of IAPs, MMERVKs and MMETns.

Supplementary Fig. 8. A shift towards the active "A" compartment at the *Zfp84* locus

a. Chromatin data at a portion of chromosome 7. The following data are displayed from top to bottom: genome co-ordinates (mm10), position of ERV elements, Hi-C Principal Component 1 (PC1) in the control (DMSO-treated) mESCs, PC1 in dTAG-13 -treated mESCs, TRIM28 ChIP-Seq data. The normalized Hi-C interaction matrix of the DMSO control and dTAG-treated samples is shown below the TRIM28 ChIP-Seq track. Note a shift of PC1 into the positive direction at loci enriched for ERV elements (highlighted with arrowheads).

b. Zoom-in of the region highlighted in panel '**a**', at the MERVK-rich *Zfp84* locus. The following data are displayed from top to bottom: genome co-ordinates (mm10), position of LTR ERV elements, gene models, TT-SLAM-Seq data (0h, and 24h dTAG-13 -treated cells), H3K27Ac ChIP-Seq (0h, and 24h dTAG-13 -treated cells), MED23 ChIP-Seq (0h, and 24h dTAG-13 -treated cells), H3K9me3 ChIP-Seq (0h, and 24h dTAG-13 -treated cells), TRIM28 ChIP-Seq. The normalized Hi-C interaction matrix of the DMSO control and dTAG-treated samples is shown below the TRIM28 ChIP-Seq track. Note a shift toward active chromatin marks and increased contact frequency at the ERV elements highlighted with arrowheads.

Supplementary Fig. 9. Cell state proportions in individual embryos reveal depletion of epiblast cells in E6.5 TRIM28 KO mouse embryos

a. (top) Cell state proportions in each embryo from the E5.5 (left), E6.5 (middle), and E7.0 (right) developmental stages analyzed in scRNA-Seq experiments. (bottom) Cell state proportions in E6.5 TRIM28 KO embryo analyzed in scRNA-Seq experiments.

b. Combined cell state proportions per embryonic stage.

Supplementary Fig. 10. Loss of pluripotency markers in TRIM28 KO blastocysts

a. The cells in the inner part of TRIM28 KO blastocysts are populated by cells that express reduced levels of OCT4 and KLF4. Displayed are representative immunofluorescence images of OCT4 and KLF4 in E3.5 wild type and TRIM28 KO blastocysts across three independent perturbation experiments with ~ 15 embryos per condition. Nuclei are stained with DAPI. Scale bars: 20µm.

b. The cells in the inner part of TRIM28 KO blastocysts are populated by cells that express reduced levels of SOX2. Displayed are representative immunofluorescence images of SOX2 in E3.5 wild type and TRIM28 KO blastocysts across three independent perturbation experiments with ~ 15 embryos per condition. Nuclei are counterstained with DAPI. Scale bars: 20µm.

Supplementary Fig. 11. Long term TRIM28 degradation induces differentiation markers in ESCs

a. 96h of TRIM28 degradation leads to an increased fraction of cells with reduced expression of the SSEA-1 pluripotency marker. Displayed are representative FACS profiles of cell populations treated for the indicated times with dTAG-13 from two biological replicates.

b. Upregulation of differentiation marker genes upon dTAG-13 treatment in mESCs. Displayed is a heatmap representation of RNA-Seq read density (TPM) Z scores across three biological replicates for the indicated genes. The annotation on the right indicates whether a gene is identified as differentially expressed at the indicated time points relative to the level at 0h.

c. Upregulation of 2C marker genes upon dTAG-13 treatment in mESCs. Displayed is a heatmap representation of RNA-Seq read density (TPM) Z scores across three biological replicates for the indicated genes. The annotation on the right indicates whether a gene is identified as differentially expressed at the indicated time points relative to the level at 0h.

Supplementary Table Legends

Table S1. List of reagents and datasets

a. RNA FISH probes

b. Primers used in the study

c. GEO accession IDs of public data used in the study

Table S2. Feature annotations

a. full length IAPs (coordinates in mm10 genome assembly coordinates)

b. full length MMERVKs (coordinates in mm10 genome assembly coordinates)

c. full length MMETns (coordinates in mm10 genome assembly coordinates)

d. Full length LINEs (coordinates in mm10 genome assembly coordinates)

e. All IAP LTRs (coordinates in mm10 genome assembly coordinates)

f. mESC enhancers (OSN constituent enhancers) (coordinates in mm10 genome assembly coordinates)

g. mESC super-enhancers (coordinates in mm10 genome assembly coordinates)

h. mESC typical enhancer (coordinates in mm10 genome assembly coordinates)

Table S3. Mass-spectrometry data

This table contains the mass spectrometry data used in Extended Data Fig. 5d and Supplementary Fig. 2c.

Table S4. TT-SLAM-Seq fold change data (genes)

This table contains the fold change values used in Fig. 1g.

Table S5. TT-SLAM-Seq fold change data (enhancers)

This table contains the fold change values used in Fig. 1i.

Table S6. Marker genes in scRNA-Seq clusters

This table contains information on the marker genes used to identify clusters in the scRNA-Seq data.

Supplementary References

1 Wernig, M. *et al.* A drug-inducible transgenic system for direct reprogramming of multiple somatic cell types. *Nature biotechnology* **26**, 916-924, doi:10.1038/nbt1483 (2008).

2 Artus, J. & Hadjantonakis, A. K. Generation of chimeras by aggregation of embryonic stem cells with diploid or tetraploid mouse embryos. *Methods in molecular biology* **693**, 37-56, doi:10.1007/978-1-60761-974-1_3 (2011).

3 Kulak, N. A., Pichler, G., Paron, I., Nagaraj, N. & Mann, M. Minimal, encapsulated proteomic-sample processing applied to copy-number estimation in eukaryotic cells. *Nature methods* **11**, 319-324, doi:10.1038/nmeth.2834 (2014).

4 Ni, Y. *et al.* Mutations in NDUFS1 Cause Metabolic Reprogramming and Disruption of the Electron Transfer. *Cells* **8**, doi:10.3390/cells8101149 (2019).

5 Martens, L. *et al.* PRIDE: the proteomics identifications database. *Proteomics* **5**, 3537-3545, doi:10.1002/pmic.200401303 (2005).

6 Orlando, D. A. *et al.* Quantitative ChIP-Seq normalization reveals global modulation of the epigenome. *Cell reports* **9**, 1163-1170, doi:10.1016/j.celrep.2014.10.018 (2014).

7 Rao, S. S. *et al.* A 3D map of the human genome at kilobase resolution reveals principles of chromatin looping. *Cell* **159**, 1665-1680, doi:10.1016/j.cell.2014.11.021 (2014).

8 Basu, S. *et al.* Unblending of Transcriptional Condensates in Human Repeat Expansion Disease. *Cell* **181**, 1062-1079 e1030, doi:10.1016/j.cell.2020.04.018 (2020).

9 Henninger, J. E. *et al.* RNA-Mediated Feedback Control of Transcriptional Condensates. *Cell* **184**, 207-225 e224, doi:10.1016/j.cell.2020.11.030 (2021).

10 Huo, X. *et al.* The Nuclear Matrix Protein SAFB Cooperates with Major Satellite RNAs to Stabilize Heterochromatin Architecture Partially through Phase Separation. *Molecular cell* **77**, 368-383 e367, doi:10.1016/j.molcel.2019.10.001 (2020).

11 Martin, M. Cutadapt removes adapter sequences from high-throughput sequencing reads. *2011* **17**, 3, doi:10.14806/ej.17.1.200 (2011).

12 Dobin, A. *et al.* STAR: ultrafast universal RNA-seq aligner. *Bioinformatics* **29**, 15-21, doi:10.1093/bioinformatics/bts635 (2013).

13 Pertea, M. *et al.* StringTie enables improved reconstruction of a transcriptome from RNA-seq reads. *Nature biotechnology* **33**, 290-295, doi:10.1038/nbt.3122 (2015).

14 Ji, X. *et al.* Chromatin proteomic profiling reveals novel proteins associated with histone-marked genomic regions. *Proceedings of the National Academy of Sciences of the United States of America* **112**, 3841-3846, doi:10.1073/pnas.1502971112 (2015).

15 Elsasser, S. J., Noh, K. M., Diaz, N., Allis, C. D. & Banaszynski, L. A. Histone H3.3 is required for endogenous retroviral element silencing in embryonic stem cells. *Nature* **522**, 240-244, doi:10.1038/nature14345 (2015).

16 Bulut-Karslioglu, A. *et al.* Suv39h-dependent H3K9me3 marks intact retrotransposons and silences LINE elements in mouse embryonic stem cells. *Molecular cell* **55**, 277-290, doi:10.1016/j.molcel.2014.05.029 (2014).

17 Whyte, W. A. *et al.* Master transcription factors and mediator establish super-enhancers at key cell identity genes. *Cell* **153**, 307-319, doi:10.1016/j.cell.2013.03.035 (2013).

18 Li, H. & Durbin, R. Fast and accurate short read alignment with Burrows-Wheeler transform. *Bioinformatics* **25**, 1754-1760, doi:10.1093/bioinformatics/btp324 (2009).

19 McKenna, A. *et al.* The Genome Analysis Toolkit: a MapReduce framework for analyzing next-generation DNA sequencing data. *Genome research* **20**, 1297-1303, doi:10.1101/gr.107524.110 (2010).

20 Zhang, Y. *et al.* Model-based analysis of ChIP-Seq (MACS). *Genome biology* **9**, R137, doi:10.1186/gb-2008-9-9-r137 (2008).

21 Kharchenko, P. V., Tolstorukov, M. Y. & Park, P. J. Design and analysis of ChIP-seq experiments for DNA-binding proteins. *Nature biotechnology* **26**, 1351-1359, doi:10.1038/nbt.1508 (2008).

22 Love, M. I., Huber, W. & Anders, S. Moderated estimation of fold change and dispersion for RNA-seq data with DESeq2. *Genome biology* **15**, 550, doi:10.1186/s13059-014-0550-8 (2014).

23 Heinz, S. *et al.* Simple combinations of lineage-determining transcription factors prime cis-regulatory elements required for macrophage and B cell identities. *Molecular cell* **38**, 576-589, doi:10.1016/j.molcel.2010.05.004 (2010).

24 Jurka, J. *et al.* Repbase Update, a database of eukaryotic repetitive elements. *Cytogenet Genome Res* **110**, 462-467, doi:10.1159/000084979 (2005).

25 Gu, Z., Eils, R., Schlesner, M. & Ishaque, N. EnrichedHeatmap: an R/Bioconductor package for comprehensive visualization of genomic signal associations. *BMC genomics* **19**, 234, doi:10.1186/s12864-018-4625-x (2018).

26 Hnisz, D. *et al.* Super-enhancers in the control of cell identity and disease. *Cell* **155**, 934-947, doi:10.1016/j.cell.2013.09.053 (2013).

27 Durinck, S., Spellman, P. T., Birney, E. & Huber, W. Mapping identifiers for the integration of genomic datasets with the R/Bioconductor package biomaRt. *Nature protocols* **4**, 1184-1191, doi:10.1038/nprot.2009.97 (2009).

28 Korotkevich, G., Sukhov, V. & Sergushichev, A. Fast gene set enrichment analysis. *bioRxiv*, 060012, doi:10.1101/060012 (2019).

29 Pockrandt, C., Alzamel, M., Iliopoulos, C. S. & Reinert, K. GenMap: ultra-fast computation of genome mappability. *Bioinformatics* **36**, 3687-3692, doi:10.1093/bioinformatics/btaa222 (2020).

30 Lawrence, M., Gentleman, R. & Carey, V. rtracklayer: an R package for interfacing with genome browsers. *Bioinformatics* **25**, 1841-1842, doi:10.1093/bioinformatics/btp328 (2009).

31 Ramirez, F., Dundar, F., Diehl, S., Gruning, B. A. & Manke, T. deepTools: a flexible platform for exploring deep-sequencing data. *Nucleic acids research* **42**, W187-191, doi:10.1093/nar/gku365 (2014).

32 Flyamer, I. M., Illingworth, R. S. & Bickmore, W. A. Coolpup.py: versatile pile-up analysis of Hi-C data. *Bioinformatics* **36**, 2980-2985, doi:10.1093/bioinformatics/btaa073 (2020).

33 McLeay, R. C. & Bailey, T. L. Motif Enrichment Analysis: a unified framework and an evaluation on ChIP data. *BMC Bioinformatics* **11**, 165, doi:10.1186/1471-2105-11-165 (2010).

34 Grosswendt, S. *et al.* Epigenetic regulator function through mouse gastrulation. *Nature* **584**, 102-108, doi:10.1038/s41586-020-2552-x (2020).

35 Krueger, F. & Andrews, S. R. SNPsplit: Allele-specific splitting of alignments between genomes with known SNP genotypes. *F1000Res* **5**, 1479, doi:10.12688/f1000research.9037.2 (2016).

36 Stuart, T. *et al.* Comprehensive Integration of Single-Cell Data. *Cell* **177**, 1888-1902 e1821, doi:10.1016/j.cell.2019.05.031 (2019).

37 La Manno, G. *et al.* RNA velocity of single cells. *Nature* **560**, 494-498, doi:10.1038/s41586-018-0414-6 (2018).

38 Wolf, F. A., Angerer, P. & Theis, F. J. SCANPY: large-scale single-cell gene expression data analysis. *Genome biology* **19**, 15, doi:10.1186/s13059-017-1382-0 (2018).

Supplementary Fig. 1

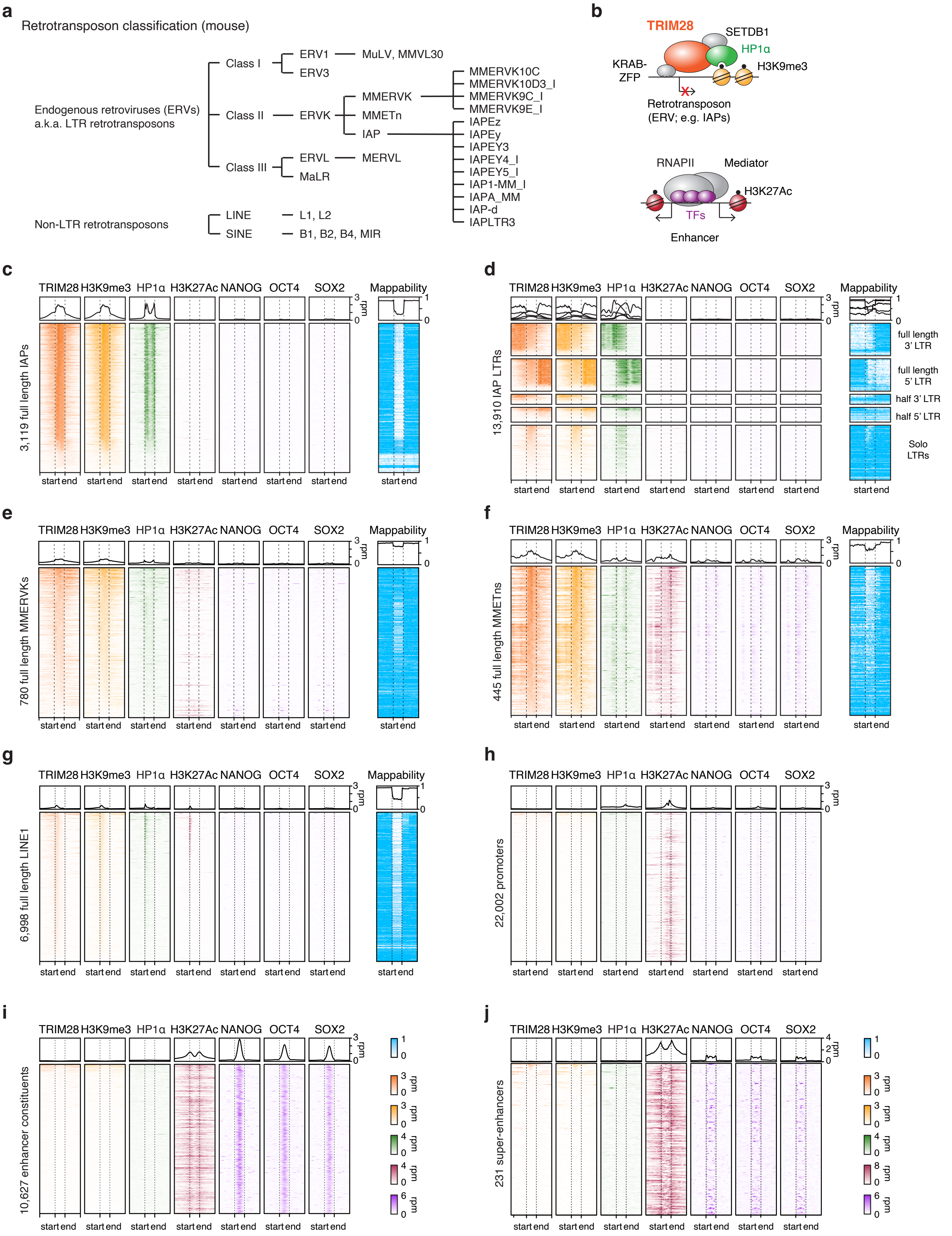

Supplementary Fig. 2

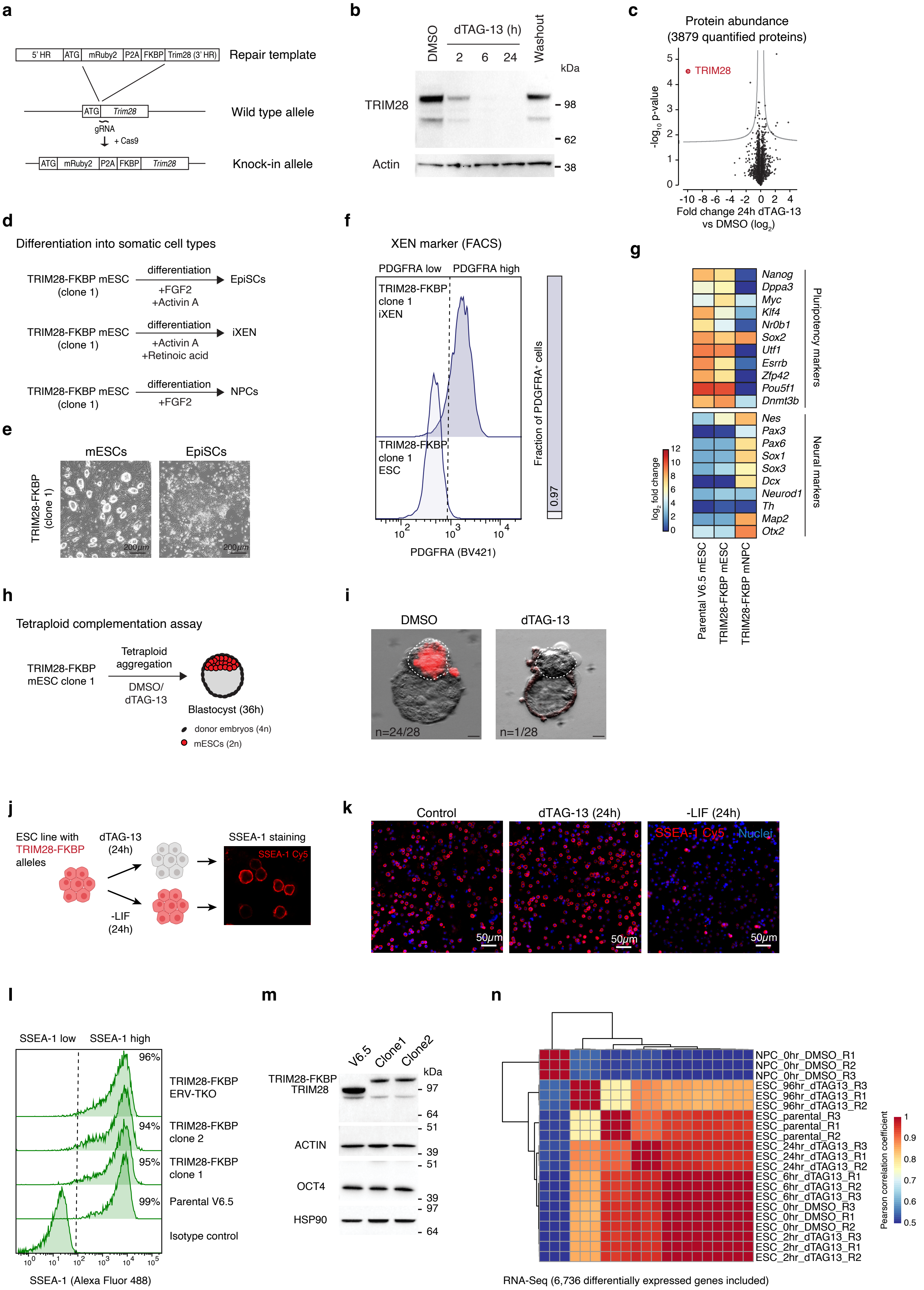

Supplementary Fig. 3

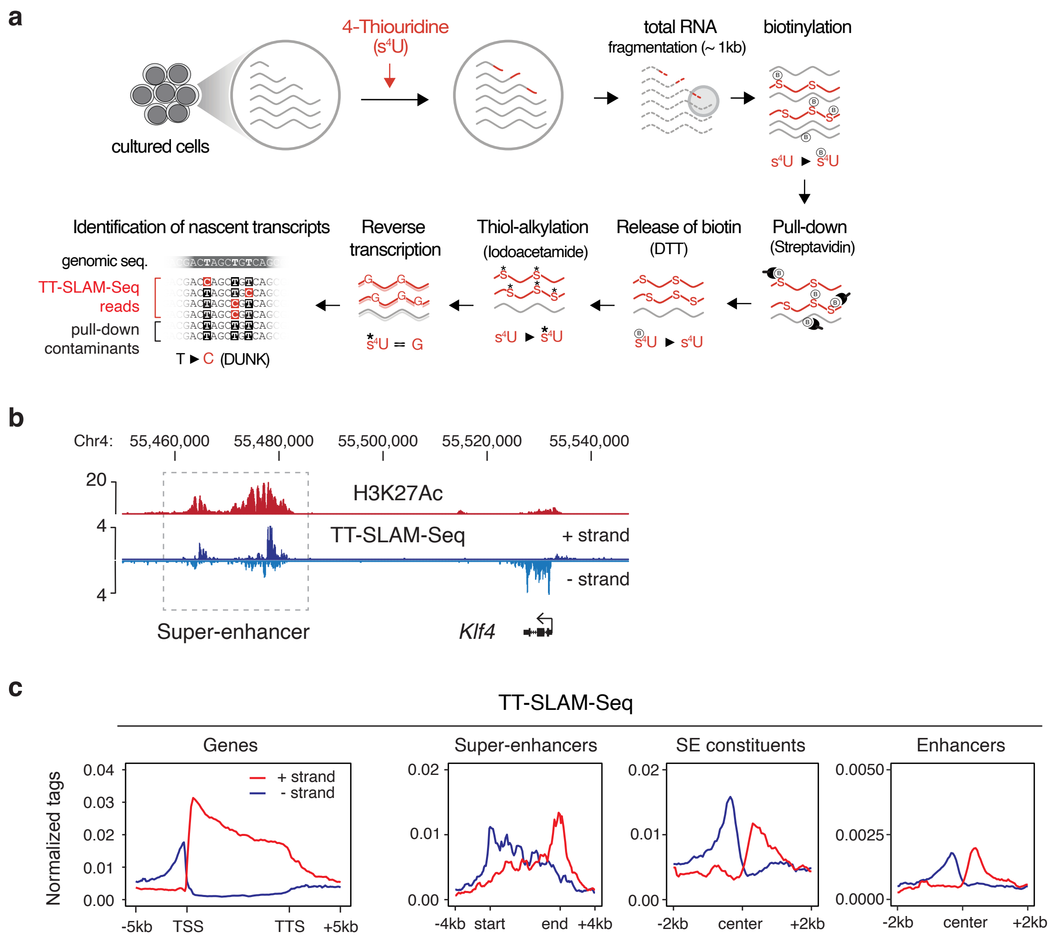

Supplementary Fig. 4

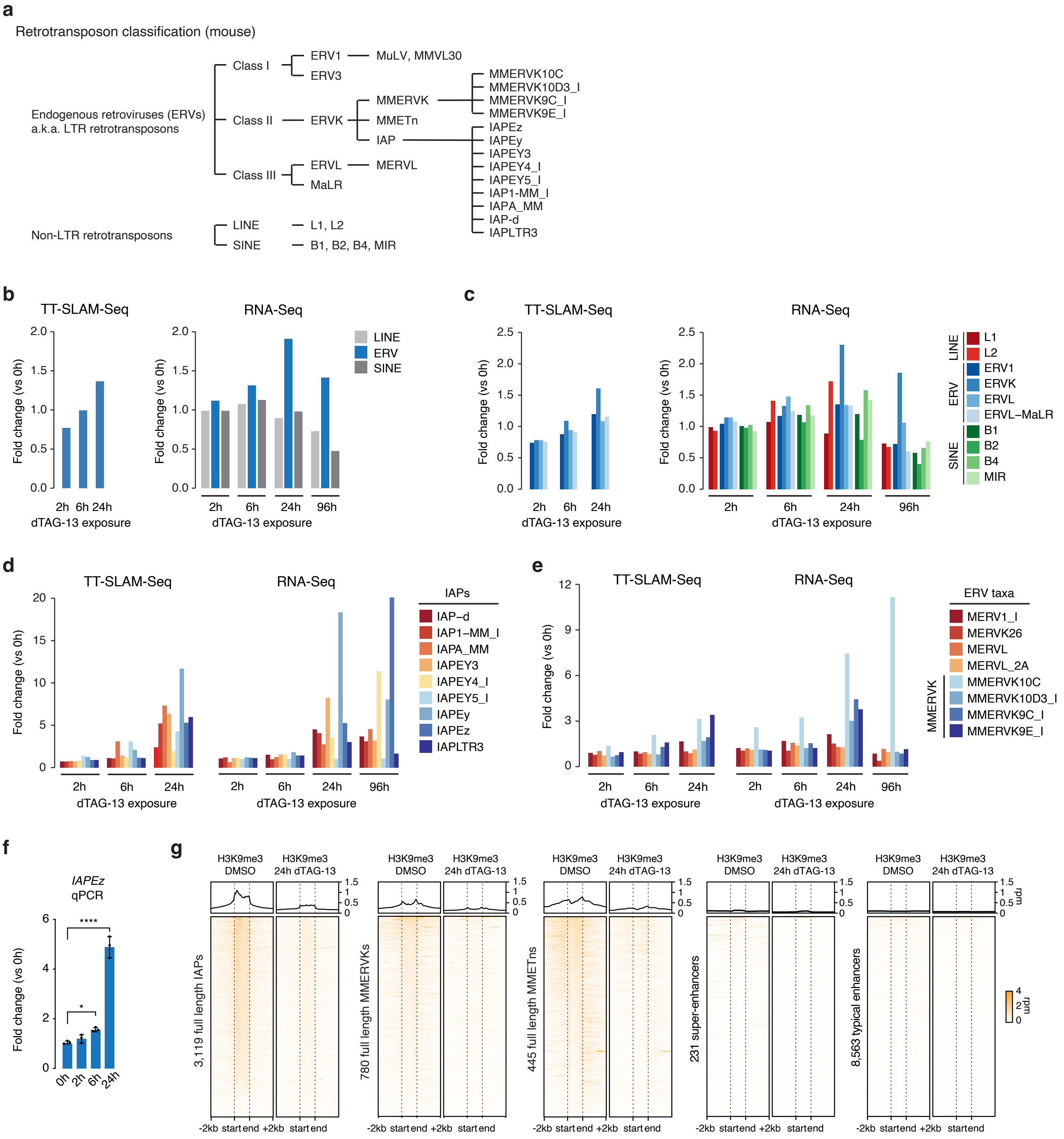

Supplementary Fig. 5

a

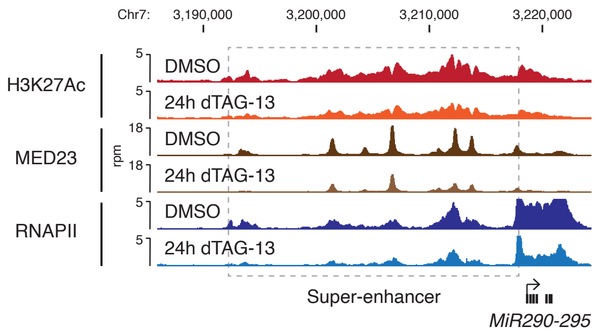

b

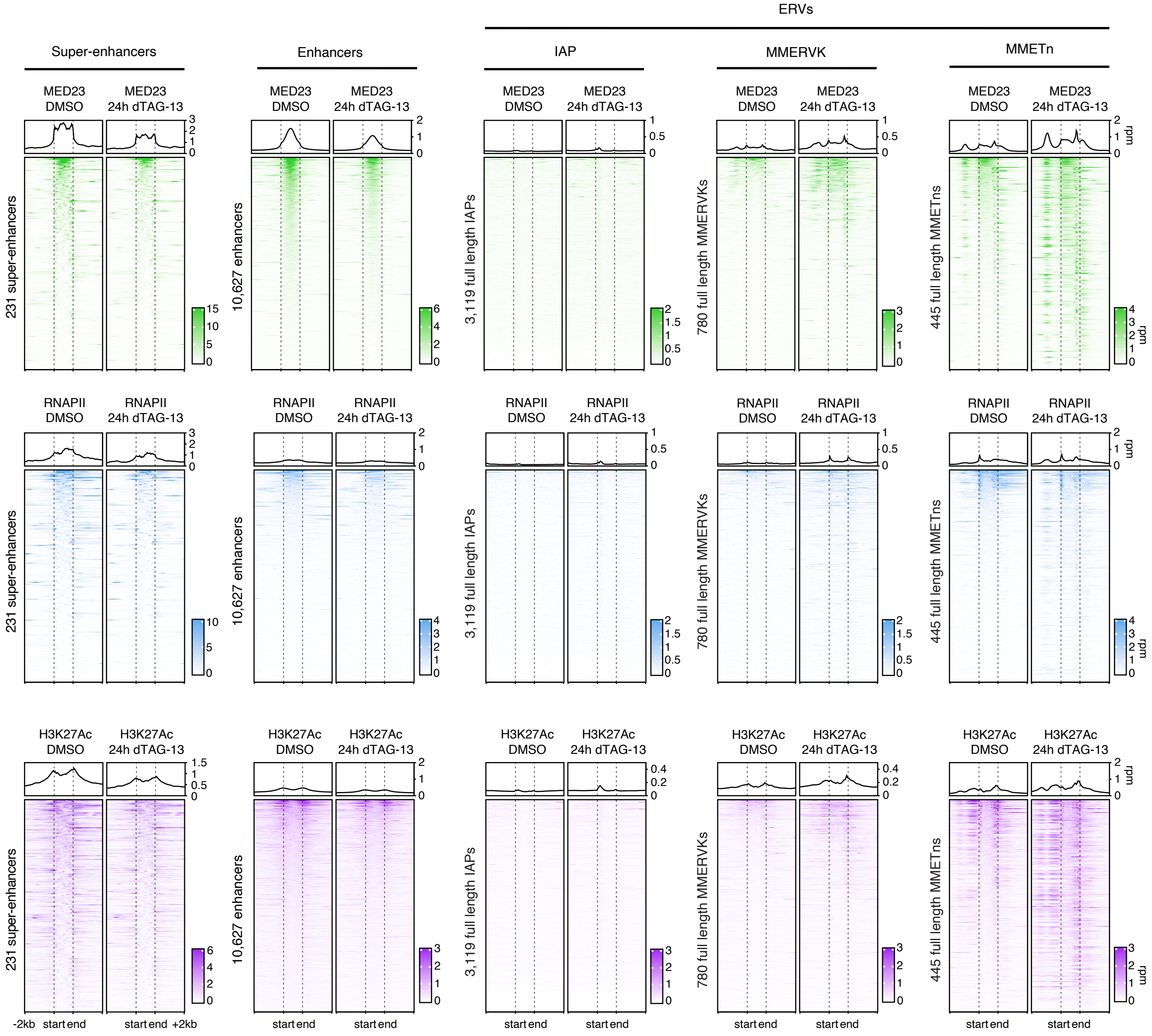

Supplementary Fig. 6

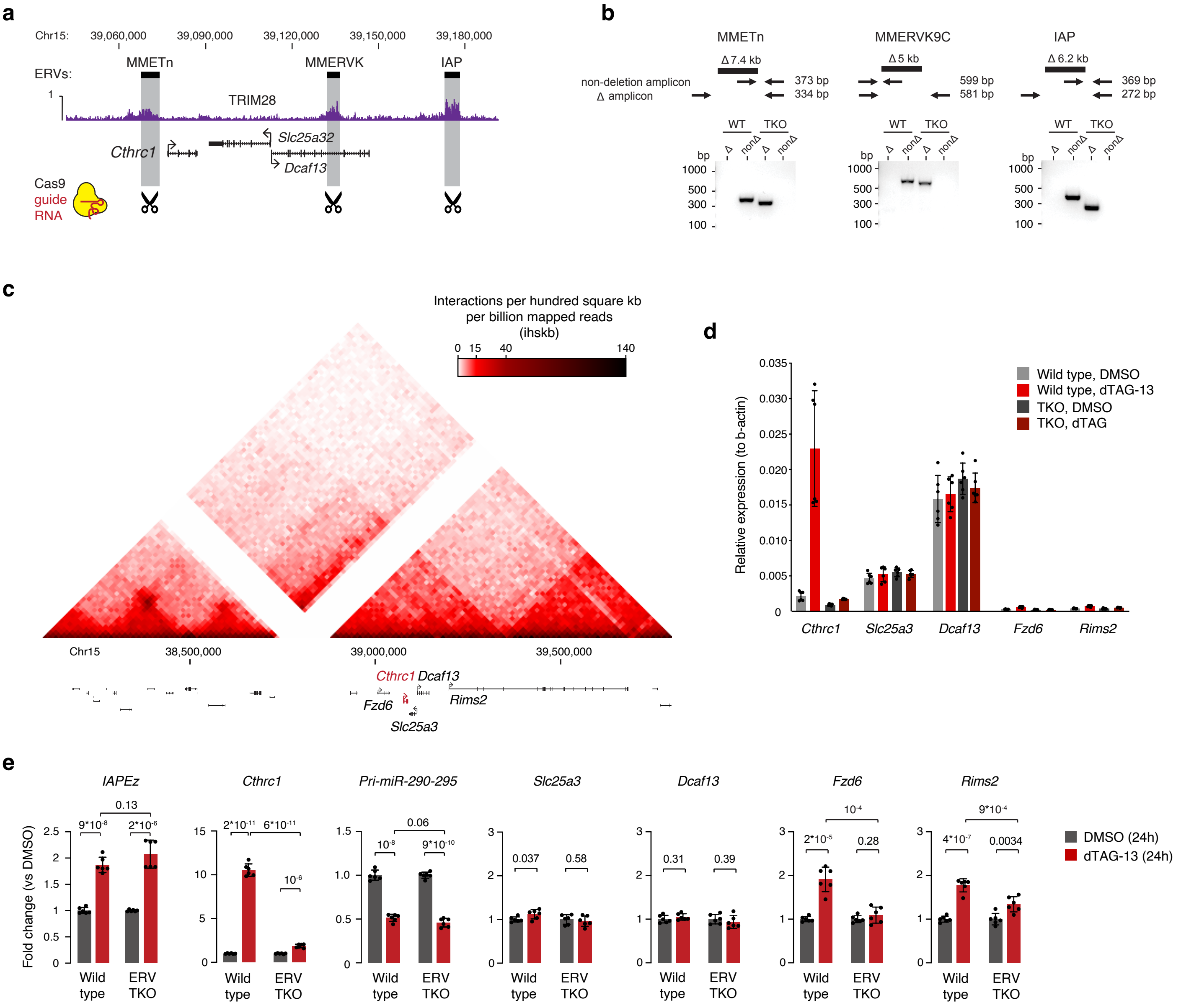

Supplementary Fig. 7

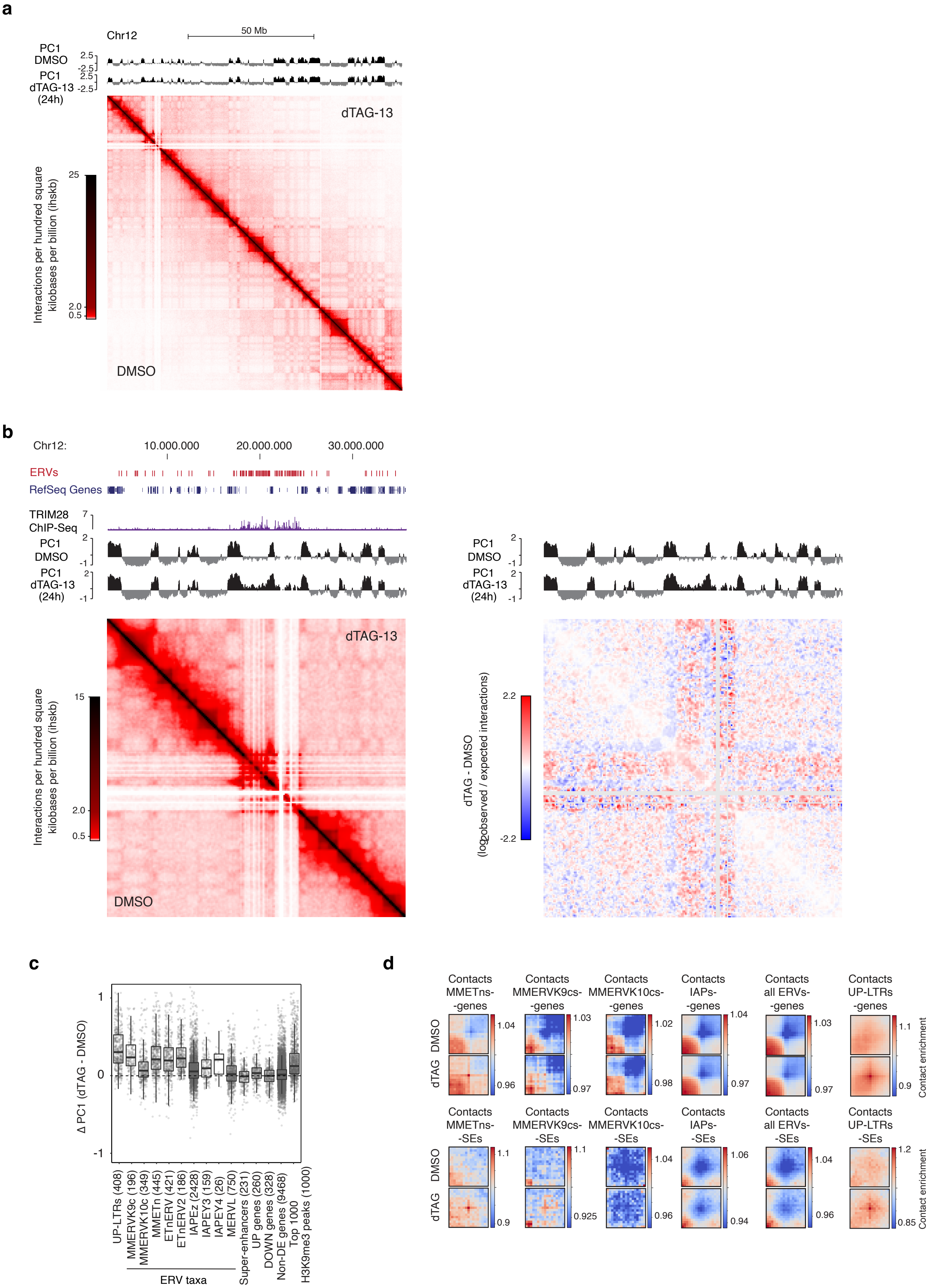

Supplementary Fig. 8

a

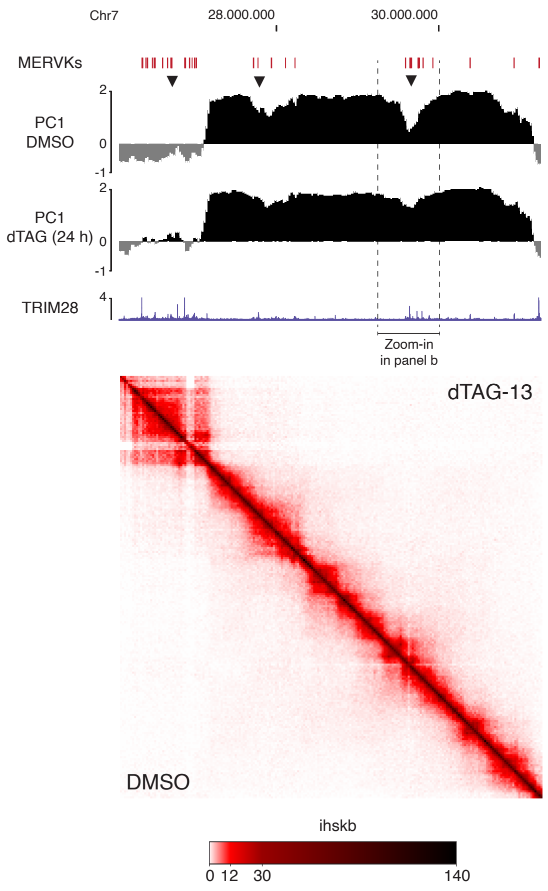

b

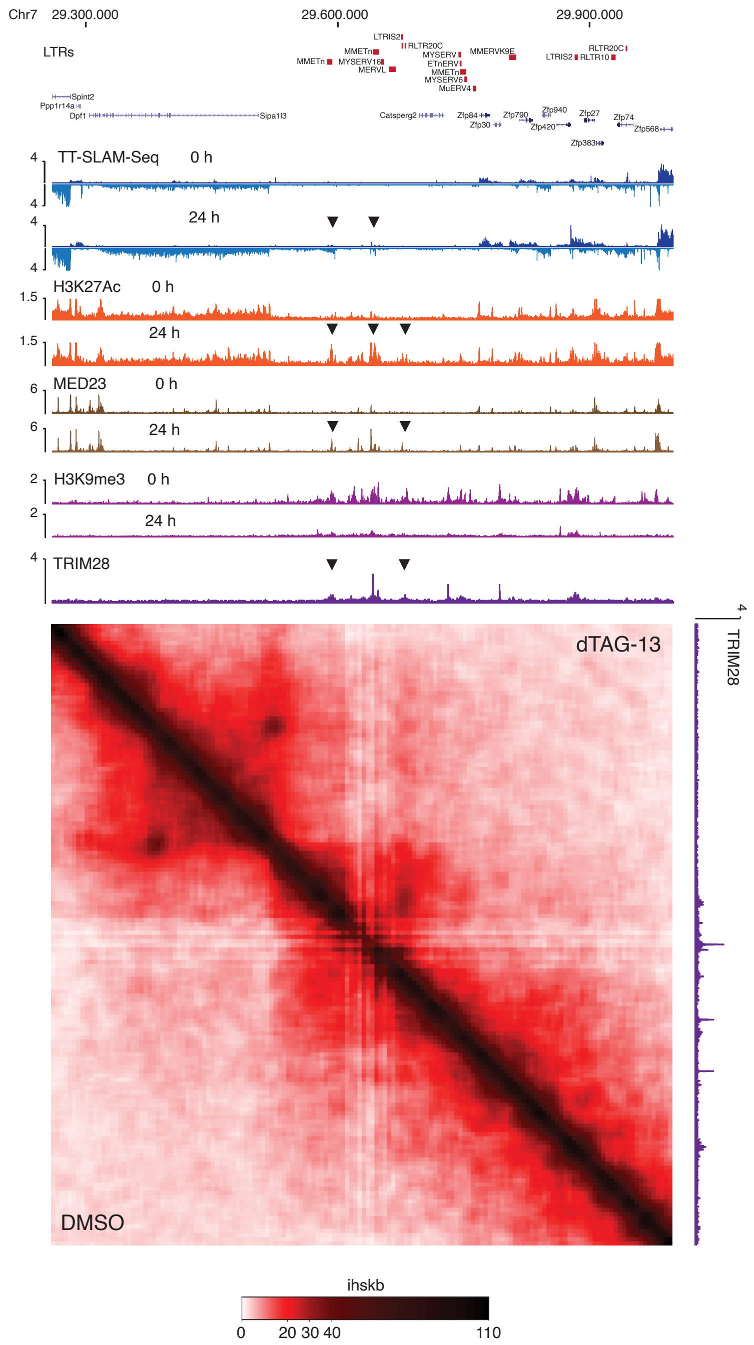

Supplementary Fig. 9

a

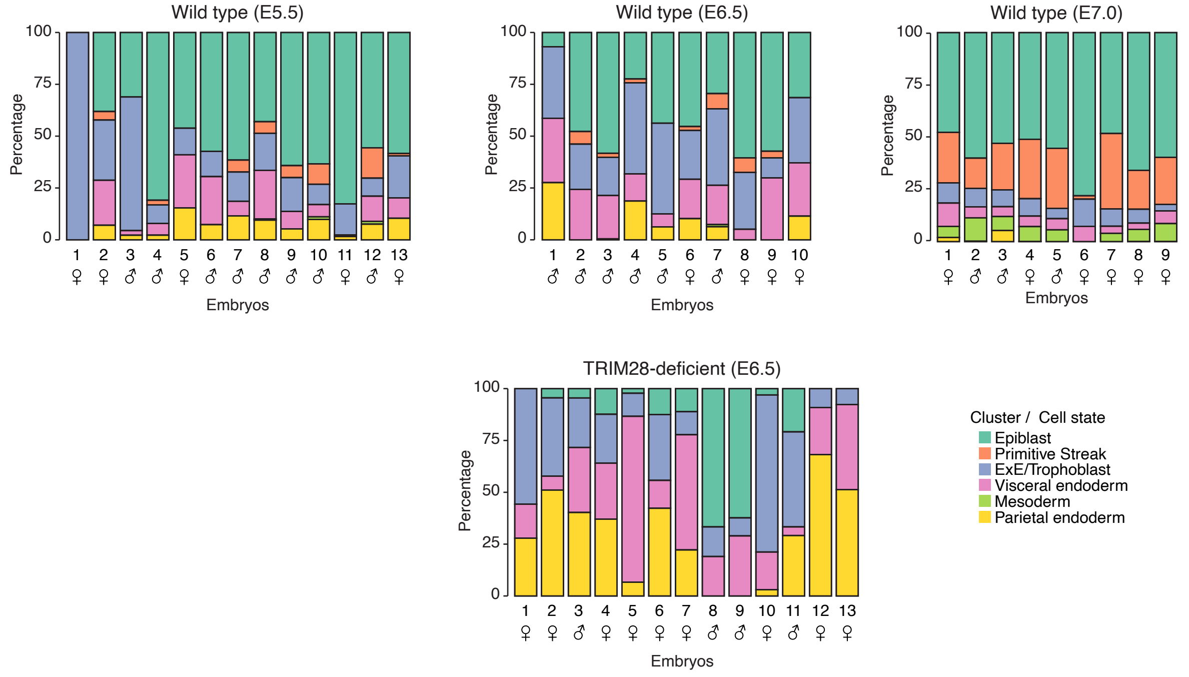

b

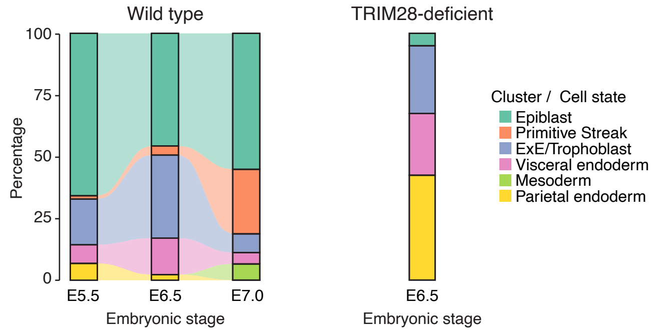

Supplementary Fig. 10

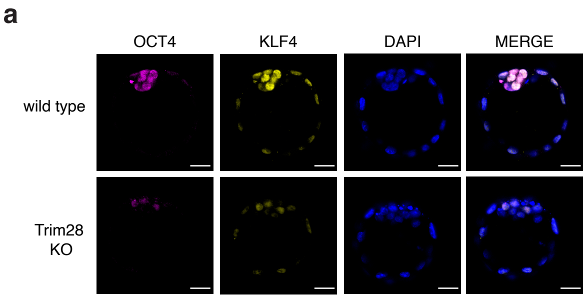

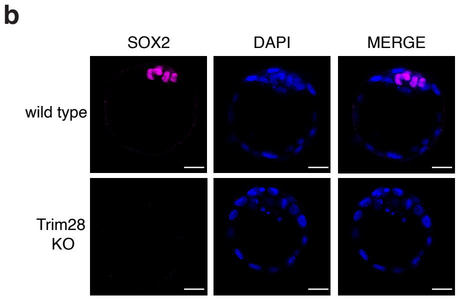

Supplementary Fig. 11

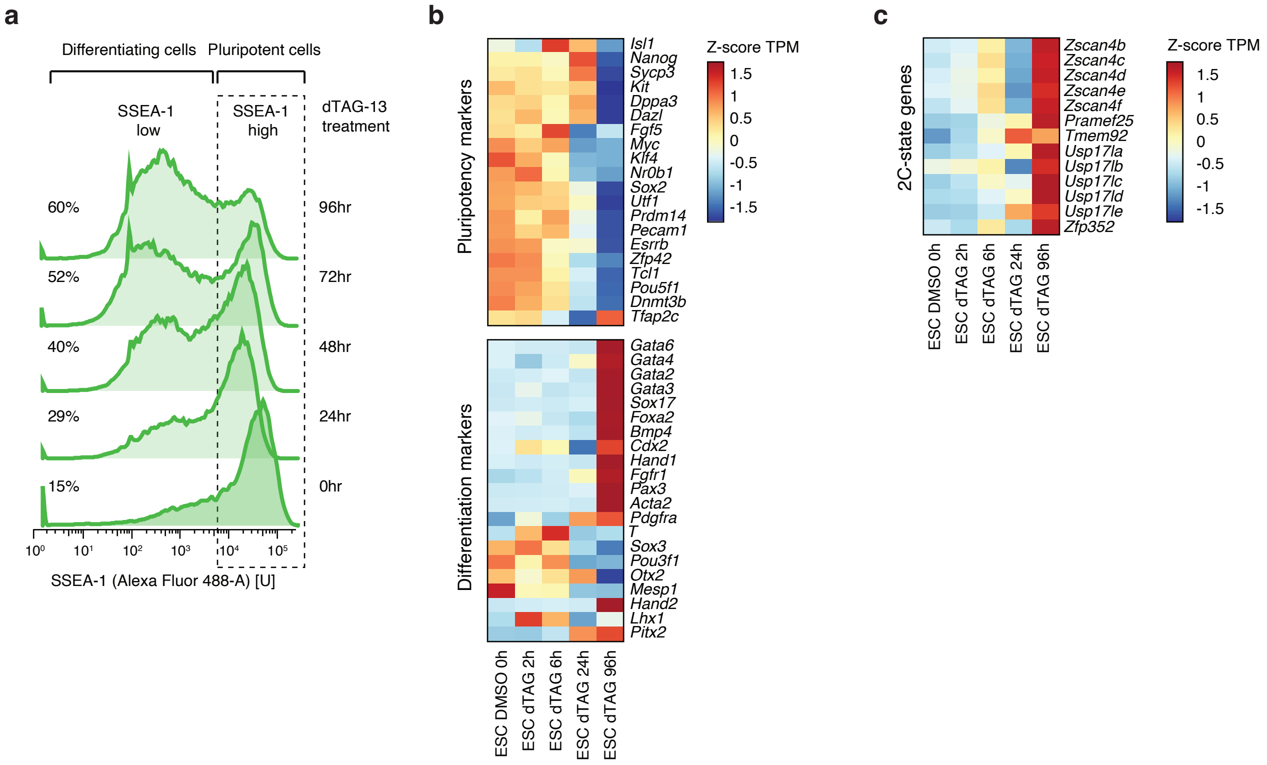

Supplement: Supplementary file 1 — Supplementary methods, figure legends, table legends, references and figures. [file 41588_2022_1132_MOESM1_ESM.pdf]
