# Supplementary material for: Hijacking of transcriptional condensates by endogenous retroviruses
Source: Nat Genet. 2022 Jul 21;54(8):1238–47. doi: 10.1038/s41588-022-01132-w (PMC9355880; doi:10.1038/s41588-022-01132-w)
Supplement: Supplementary file 2 — Reporting Summary [file 41588_2022_1132_MOESM2_ESM.pdf]

nature research

Corresponding author(s): Denes Hnisz

Last updated by author(s): May 4, 2022

Reporting Summary

Nature Research wishes to improve the reproducibility of the work that we publish. This form provides structure for consistency and transparency in reporting. For further information on Nature Research policies, see our Editorial Policies and the Editorial Policy Checklist.

Statistics

For all statistical analyses, confirm that the following items are present in the figure legend, table legend, main text, or Methods section.

n/a	Confirmed	
☐	☒	The exact sample size (*n*) for each experimental group/condition, given as a discrete number and unit of measurement
☒	☐	A statement on whether measurements were taken from distinct samples or whether the same sample was measured repeatedly
☐	☒	The statistical test(s) used AND whether they are one- or two-sided<br>*Only common tests should be described solely by name; describe more complex techniques in the Methods section.*
☒	☐	A description of all covariates tested
☐	☒	A description of any assumptions or corrections, such as tests of normality and adjustment for multiple comparisons
☐	☒	A full description of the statistical parameters including central tendency (e.g. means) or other basic estimates (e.g. regression coefficient) AND variation (e.g. standard deviation) or associated estimates of uncertainty (e.g. confidence intervals)
☐	☒	For null hypothesis testing, the test statistic (e.g. *F*, *t*, *r*) with confidence intervals, effect sizes, degrees of freedom and *P* value noted<br>*Give P values as exact values whenever suitable.*
☒	☐	For Bayesian analysis, information on the choice of priors and Markov chain Monte Carlo settings
☒	☐	For hierarchical and complex designs, identification of the appropriate level for tests and full reporting of outcomes
☐	☒	Estimates of effect sizes (e.g. Cohen's *d*, Pearson's *r*), indicating how they were calculated

Our web collection on statistics for biologists contains articles on many of the points above.

Software and code

Policy information about availability of computer code

Data collection

- Fluorescence images were collected with widefield and confocal microscopes using Zen Blue (2.3.69.1016) and Zen (black version) software (Zeiss)

- Western blot images were collected using Image Lab software (version 6.1.0 build 7) (Bio-Rad)

- Single-cell data was collected with 10X Cell Genomics Chromium System v2.0 chemistry (10X Genomics)

- FACS data was collected with FACS Diva software (BD Biosciences) and BD Aria II and BD Celesta instruments

Data analysis

- Fluorescence images were analyzed using:

Zeiss Zen Blue (2.3.69.1016)

Zeiss Zen (black version)

Fiji/ImageJ (2.1.0/1.53i)

Published MATLAB scripts (Sabari et al., 2018) were used for the analysis of RNA FISH with immunofluorescence. Custom python code was generated to curate random nuclear foci

- Fiji/ImageJ colocalization plugins (Bolte & Cordelieres, 2006, Gilles et al., 2017) were used for IAP FISH-IF analysis

- FACS data was analyzed with FlowJo (v10.7)

- GraphPad Prism (v 9.2.0) was used for statistical analysis and barplot generation.

- PALM images were reconstructed and analyzed using MTT (Sergé et al., 2008) and qSR (Andrews et al., 2018).

- For NGS data analyses, the following softwares were used:

Seurat (version 3.2.3)

cutadapt (version 2.4)

STAR (version 2.7.5a)

StringTie (version 2.0.6)

Seqtk (version 1.3-r106)

SLAM-DUNK (version 0.4.1)

DeppTools bamCoverage (version 3.4.3)

bwa mem (version 0.7.17)

samtools (version 1.10)

gatk (version 4.1.4.1)

Spp (version 1.2.2)

MACS2 (version 2.1.2)

HOMER (version 4.10)

Treeview (version 3.0)

Ame (version 5.3.0)

Cell Ranger (version 4)

velocyto (version 0.1.18)

scanpy (version 1.4.3)

pheatmap (version 1.0.12)

Coolpup.py (version 0.9.5)

All software versions and parameters are listed in the Methods.

Custom code available under : https://doi.org/10.5281/zenodo.6521914

For manuscripts utilizing custom algorithms or software that are central to the research but not yet described in published literature, software must be made available to editors and reviewers. We strongly encourage code deposition in a community repository (e.g. GitHub). See the Nature Research guidelines for submitting code & software for further information.

Data

Policy information about availability of data

All manuscripts must include a data availability statement. This statement should provide the following information, where applicable:

- Accession codes, unique identifiers, or web links for publicly available datasets
- A list of figures that have associated raw data
- A description of any restrictions on data availability

All data is available in the supplementary materials. Sequence data were deposited at GEO under the accession number GSE159468. Mass spectrometry data were deposited at ProteomeXchange under the accession ID PDX021895. Plasmids generated in the study are available at Addgene.

Field-specific reporting

Please select the one below that is the best fit for your research. If you are not sure, read the appropriate sections before making your selection.

☒ Life sciences ☐ Behavioural & social sciences ☐ Ecological, evolutionary & environmental sciences

For a reference copy of the document with all sections, see nature.com/documents/nr-reporting-summary-flat.pdf

Life sciences study design

All studies must disclose on these points even when the disclosure is negative.

Sample size	No statistical methods were used to predetermine sample sizes. Sample sizes for qPCR, Mass spectrometry, RNA-FISH/IF, IF, FACS, ChIP-seq, RNA-seq, SLAM-TT-Seq, and other sequencing technologies are consistent with current standards for sample sizes included controls in the published literature. Sample sizes are indicated in the figure panels or legends or in the Methods. For droplet experiments we imaged at least 10 independent fields of a view for each experimental condition based on current methodology in the field (Sabari et al., 2018; Boija et al., 2018).For RNA FISH with IF we imaged at least two fields of view (yielding 20+ nuclei and the number of detected foci indicated in figure panels) for each condition based on current practices in the field (Sabari et al., 2018, Boija et al., 2018).
Data exclusions	In rare instances, out of focus images were excluded in FISH-IF experiments.
Replication	All results obtained and reported in this study were reproducible across the replicates examined. The number of replicates per experiment has been described in the figures, legends, tables, main text or supplementary materials.
Randomization	Not relevant in this study. Samples were allocated as either wildtype/control group or chemical/genetic treatment group.<br>Cell culture samples for every experiment were collected without a preconceived selection strategy. Embryos were also collected without a preconceived selection strategy or priority. All experiments were performed with appropriate controls. Controls are included in the individual figure panels.
Blinding	Blinding was not relevant for the experiments. However, our analytical pipeline for each experiment followed uniform criteria applied to all samples, allowing us to analyze our data in an unbiased manner.

Reporting for specific materials, systems and methods

We require information from authors about some types of materials, experimental systems and methods used in many studies. Here, indicate whether each material, system or method listed is relevant to your study. If you are not sure if a list item applies to your research, read the appropriate section before selecting a response.

Materials & experimental systems

n/a	Involved in the study
☐	☒ Antibodies
☐	☒ Eukaryotic cell lines
☒	☐ Palaeontology and archaeology
☐	☒ Animals and other organisms
☒	☐ Human research participants
☒	☐ Clinical data
☒	☐ Dual use research of concern

Methods

n/a	Involved in the study
☐	☒ ChIP-seq
☐	☒ Flow cytometry
☒	☐ MRI-based neuroimaging

Antibodies

Antibodies used

- Immunofluorescence experiments:

TRIM28 (ab22553; 1:200, Abcam)
OCT4 (ab19857; 1:200, Abcam)
NANOG (REC-RCAB002P-F; 1:400, CosmoBio)
KLF4 (AF3158; 1:200, R&D Systems)
SOX2 (ab79351; 1:200, Abcam)
GATA6 (AF1700; 1:200, R&D Systems)
GATA4 (sc-25310; 1:200, Santa Cruz)
SOX17 (AF1924; 1:200, R&D Systems)
SSEA-1 antibody (BioLegend 125609/125608; 1:1000)
IAP-GAG (MBS8566075; 1:100, MyBioSource)
MED1 (IF: Abcam ab64965 lot GR3326781-1)
MED23 (IF: Bethyl A300-425A lot no. 1)
Pol II-CTD (IF: Abcam ab817 lot GR3216482-4)
NRF1 (IF: Abcam ab55744 lot GR3334322-3)
NFYA (IF: Santa Cruz sc-17753 X lot F0916)
Goat anti-Rabbit-Alexa 488 (Thermo A11008 lot 2179202)
Goat anti-Mouse-Alexa 488 (Thermo A11001 lot 2189178)
Donkey anti-Rabbit-Alexa 488 (A21206, Invitrogen)
Donkey anti-Rabbit-Alexa647 (711-605-152, Jackson Immuno)
Donkey anti-Mouse-Alexa 488 (A21202, Invitrogen)
Donkey anti-Mouse-Alexa 647 (715-605-150, Jackson Immuno)
Donkey anti-Goat-Alexa 594 (A11058, Invitrogen)
Donkey anti-Goat-Alexa 647 (705-605-147, Jackson Immuno)

April 2020

-Western blotting experiments:

TRIM28 (ab22553; 1:500, Abcam)
ACTB (ab8226; 1:1000, Abcam)
OCT4 (ab19857; 1:500, Abcam)
OCT4 (sc-5279; 1:500, Santa Cruz)
HSP90 (BD610419; 1:4000, BD Bioisceinces)
SOX2 (ab79351; 1:500, Abcam)
HRP secondary (Jackson Immuno #115-035-174, 211-032-171)

- FACS experiments:

SSEA-1 antibody (BioLegend 125609/125608; 1:1000)
PDGFRA (BioLegend 135923; 1:1000)

-ChIP experiments:

Spike-in antibody (61686, Active Motif)
H3K9me3 (ab8898, Abcam)
H3K27ac (ab4729, Abcam)
Pol II-CTD (8WG16, BioLegend)
MED23 (A300-425A, Bethyl Lab)

Validation

Antibodies used in immunofluorescence experiments were validated by comparing WT mESCs and mouse embryos.
Antibody validation for IAP-GAG was performed with wild type 2C-8C stage mouse embryos.

All antibodies are validated by the provider and cited in numerous publications.

-Western blotting experiments:

TRIM28 (ab22553; 1:500, Abcam) - mouse
https://www.abcam.com/kap1-antibody-20c1-ab22553.html

ACTB (ab8226; 1:1000, Abcam) - mouse
https://www.abcam.com/beta-actin-antibody-mabcam-8226-loading-control-ab8226.html

OCT4 (ab19857; 1:500, Abcam) - mouse
https://www.abcam.com/oct4-antibody-ab19857.html

OCT4 (sc-5279; 1:500, Santa Cruz) - mouse
https://www.scbt.com/p/oct-3-4-antibody-c-10

HSP90 (BD610419; 1:4000, BD Bioisceinces) - mouse
https://www.fishersci.com/shop/products/anti-hsp90-clone-68-bd-2/BDB610419

SOX2 (ab79351; 1:500, Abcam) - mouse
https://www.abcam.com/sox2-antibody-9-9-3-ab79351.html

- FACS experiments:

SSEA-1 antibody (BioLegend 125609/125608; 1:1000) - mouse
https://www.biolegend.com/ja-jp/products/alexa-fluor-488-anti-mouse-human-cd15-ssea-1-antibody-4820
https://www.biolegend.com/ja-jp/products/alexa-fluor-647-anti-mouse-human-cd15-ssea-1-antibody-4819

PDGFRA (BioLegend 135923; 1:1000) - mouse
https://www.biolegend.com/ja-jp/products/brilliant-violet-421-anti-mouse-cd140a-antibody-17921

-ChIP experiments:

Spike-in antibody (61686, Active Motif)
https://www.activemotif.com/catalog/1091/chip-normalization

H3K9me3 (ab8898, Abcam) - mouse
https://www.abcam.com/histone-h3-tri-methyl-k9-antibody-chip-grade-ab8898.html

H3K27ac (ab4729, Abcam) - mouse
https://www.abcam.com/histone-h3-acetyl-k27-antibody-chip-grade-ab4729.html

Pol II-CTD (8WG16, BioLegend) - mouse
https://www.biolegend.com/en-us/punchout/search-results/purified-anti-rna-polymerase-ii-rpb1-antibody-11666

MED23 (A300-425A, Bethyl Lab) - mouse
https://www.fortislife.com/products/primary-antibodies/rabbit-anti-crsp3-antibody/BETHYL-A300-423

Eukaryotic cell lines

Policy information about cell lines

Cell line source(s)	- V6.5 mouse embryonic stem cells (mESCs), source: Konrad Hochedlinger lab<br><br>- HEK293T, source: ATCC, Identifier: CRL-3216<br><br>- Trim28-FKBP mESC, source: This paper (see Methods "Generation of the TRIM28-FKBP ESC line")<br><br>- Trim28-FKBP mouse iPSC, source: This paper (see Methods "Generation of the TRIM28-FKBP iPSC line")<br><br>- Trim28-FKBP mouse iPSC, inactivation of GFP (Nanog::GFP), source: This paper (see Methods "Inactivation of NANOG::GFP in the TRIM28-FKBP iPSC line")<br><br>- Trim28-FKBP mESCs IAPEz knockdown, source: This paper (see Methods "Generation of shRNA knockdown mESC line for IAPEz and IAPEz/MMERVK10c/ /MMERVK9c/ MMETn")<br><br>- Trim28-FKBP mESCs ERV knockdown, source: This paper (see Methods "Generation of shRNA knockdown mESC line for IAPEz and IAPEz/MMERVK10c/ /MMERVK9c/ MMETn")<br><br>- Trim28-FKBP mESCs ERV-TKO, cource: This paper (see Methods "Deletion of ERVs in the TRIM28-FKBP mESC line")<br><br>- mESCs inducible ERVs, source: This paper (see Methods "Integration of PiggyBac transposon encoding Dox-inducible ERVs")<br><br>- Trim28-FKBP mEpiSCs, source: This paper (see Methods "Differentiation of TRIM28-FKBP mESCs to EpiSCs (epiblast stem cells)")<br><br>- Trim28-FKBP mNPCs, source: This paper (see Methods "Differentiation of TRIM28-FKBP mESCs to iXEN (induced extra-embryonic stem cells)", "Differentiation of TRIM28-FKBP mESCs to NPCs (neural progenitor cells)")<br><br>- Trim28-FKBP iXEN, source: This paper (see Methods "Differentiation of TRIM28-FKBP mESCs to iXEN (induced extra-embryonic stem cells)")
Authentication	The identity of HEK293T, parental V6.5 mESCs and iPSCs, and all cell lines derived from them has been validated using morphological characteristics, qPCRs, FACS, immunofluorescence, RNA-seq, and marker gene expression (where applicable), but have not been authenticated.
Mycoplasma contamination	All cell lines are negative for mycoplasma contamination and were regularly tested throughout the study.
Commonly misidentified lines<br>(See ICLAC register)	No commonly misidentified cell lines were used in this study.

Animals and other organisms

Policy information about studies involving animals; ARRIVE guidelines recommended for reporting animal research

Laboratory animals	Oocytes were isolated from B6D2F1 strain female mice (age 7 to 9 weeks, Envigo), sperm was isolated from B6/CAST F1 male mice (>2months of age) which were generated by breeding C57BL/6J strain female mice with CAST/EiJ strain males. Blastocysts were transferred into Hsd:ICR (CD-1) strain female mice of age 9-12 weeks (21-25g, Envigo) which had been mated with Vasectomized SW strain males of >1 age (Envigo). All mice were kept under SPF-conditions in individually ventilated cages at a temperature of 22 +/- 2 degree celcius and a humidity of 55% +/- 10% with a 12hr light/dark cycle (6am-6pm).
Wild animals	Wild animals were not involved in this study.
Field-collected samples	No samples were collected from the field.
Ethics oversight	All procedures follow strict animal welfare guidelines as approved by the Max Planck Institute for Molecular Genetics (G0247/13-SGr1) and LAGeSO Berlin; Harvard University license numbers IACUC protocol (28-21).

Note that full information on the approval of the study protocol must also be provided in the manuscript.

ChIP-seq

Data deposition

☒ Confirm that both raw and final processed data have been deposited in a public database such as GEO.

☒ Confirm that you have deposited or provided access to graph files (e.g. BED files) for the called peaks.

Data access links<br>*May remain private before publication.*	Datasets generated in this study have been deposited in the Gene Expression Omnibus under accession number GSE159468

Files in database submission

PROCESSED DATA FILES

H3K27Ac_ESC_DMSO_rep1.bw
H3K27Ac_ESC_DMSO_rep2.bw
H3K27Ac_ESC_DMSO_rep3.bw
H3K27Ac_ESC_dTAG_24h_rep1.bw
H3K27Ac_ESC_dTAG_24h_rep2.bw
H3K27Ac_ESC_dTAG_24h_rep3.bw
H3K9me3_ESC_DMSO_rep1.bw
H3K9me3_ESC_DMSO_rep2.bw
H3K9me3_ESC_DMSO_rep3.bw
H3K9me3_ESC_dTAG_24h_rep1.bw
H3K9me3_ESC_dTAG_24h_rep2.bw
H3K9me3_ESC_dTAG_24h_rep3.bw
input_ESC_DMSO_rep1.bw
input_ESC_DMSO_rep2.bw
input_ESC_DMSO_rep3.bw
input_ESC_dTAG_24h_rep1.bw
input_ESC_dTAG_24h_rep2.bw
input_ESC_dTAG_24h_rep3.bw
Pol2_ESC_DMSO_rep1.bw
Pol2_ESC_DMSO_rep2.bw
Pol2_ESC_dTAG_24h_rep1.bw
Pol2_ESC_dTAG_24h_rep2.bw
Med23_ESC_DMSO_rep1.bw
Med23_ESC_DMSO_rep2.bw
Med23_ESC_dTAG_24h_rep1.bw
Med23_ESC_dTAG_24h_rep2.bw
input_ESC_DMSO_Pol2.bw
input_ESC_dTAG_24h_Pol2.bw
input_ESC_DMSO_Med23.bw
input_ESC_dTAG_24h_Med23.bw
H3K27Ac_ESC_DMSO_rep1_narrowPeak.bed.gz
H3K27Ac_ESC_DMSO_rep2_narrowPeak.bed.gz
H3K27Ac_ESC_DMSO_rep3_narrowPeak.bed.gz
H3K27Ac_ESC_dTAG_24h_rep1_narrowPeak.bed.gz
H3K27Ac_ESC_dTAG_24h_rep2_narrowPeak.bed.gz
H3K27Ac_ESC_dTAG_24h_rep3_narrowPeak.bed.gz
H3K9me3_ESC_DMSO_rep1_broadPeak.bed.gz
H3K9me3_ESC_DMSO_rep2_broadPeak.bed.gz
H3K9me3_ESC_DMSO_rep3_broadPeak.bed.gz
H3K9me3_ESC_dTAG_24h_rep1_broadPeak.bed.gz
H3K9me3_ESC_dTAG_24h_rep2_broadPeak.bed.gz
H3K9me3_ESC_dTAG_24h_rep3_broadPeak.bed.gz
Pol2_ESC_DMSO_rep1_narrowPeak.bed.gz
Pol2_ESC_DMSO_rep2_narrowPeak.bed.gz
Pol2_ESC_dTAG_24h_rep1_narrowPeak.bed.gz
Pol2_ESC_dTAG_24h_rep2_narrowPeak.bed.gz
Med23_ESC_DMSO_rep1_narrowPeak.bed.gz
Med23_ESC_DMSO_rep2_narrowPeak.bed.gz
Med23_ESC_dTAG_24h_rep1_narrowPeak.bed.gz
Med23_ESC_dTAG_24h_rep2_narrowPeak.bed.gz

RAW DATA FILES
H3K27Ac_ESC_DMSO_rep1_R1.fastq.gz
H3K27Ac_ESC_DMSO_rep2_R1.fastq.gz
H3K27Ac_ESC_DMSO_rep3_R1.fastq.gz
H3K27Ac_ESC_dTAG_24h_rep1_R1.fastq.gz
H3K27Ac_ESC_dTAG_24h_rep2_R1.fastq.gz
H3K27Ac_ESC_dTAG_24h_rep3_R1.fastq.gz
H3K9me3_ESC_DMSO_rep1_R1.fastq.gz
H3K9me3_ESC_DMSO_rep2_R1.fastq.gz
H3K9me3_ESC_DMSO_rep3_R1.fastq.gz
H3K9me3_ESC_dTAG_24h_rep1_R1.fastq.gz
H3K9me3_ESC_dTAG_24h_rep2_R1.fastq.gz
H3K9me3_ESC_dTAG_24h_rep3_R1.fastq.gz
input_ESC_DMSO_rep1_R1.fastq.gz
input_ESC_DMSO_rep2_R1.fastq.gz
input_ESC_DMSO_rep3_R1.fastq.gz
input_ESC_dTAG_24h_rep1_R1.fastq.gz
input_ESC_dTAG_24h_rep2_R1.fastq.gz
input_ESC_dTAG_24h_rep3_R1.fastq.gz
Pol2_ESC_DMSO_rep1_R1.fastq.gz
Pol2_ESC_DMSO_rep2_R1.fastq.gz
Pol2_ESC_dTAG_24h_rep1_R1.fastq.gz

Pol2_ESC_dTAG_24h_rep2_R1.fastq.gz
Med23_ESC_DMSO_rep1_R1.fastq.gz
Med23_ESC_DMSO_rep2_R1.fastq.gz
Med23_ESC_dTAG_24h_rep1_R1.fastq.gz
Med23_ESC_dTAG_24h_rep2_R1.fastq.gz
input_ESC_DMSO_Pol2_R1.fastq.gz
input_ESC_dTAG_24h_Pol2_R1.fastq.gz
input_ESC_DMSO_Med23_R1.fastq.gz
input_ESC_dTAG_24h_Med23_R1.fastq.gz
H3K27Ac_ESC_DMSO_rep1_R2.fastq.gz
H3K27Ac_ESC_DMSO_rep2_R2.fastq.gz
H3K27Ac_ESC_DMSO_rep3_R2.fastq.gz
H3K27Ac_ESC_dTAG_24h_rep1_R2.fastq.gz
H3K27Ac_ESC_dTAG_24h_rep2_R2.fastq.gz
H3K27Ac_ESC_dTAG_24h_rep3_R2.fastq.gz
H3K9me3_ESC_DMSO_rep1_R2.fastq.gz
H3K9me3_ESC_DMSO_rep2_R2.fastq.gz
H3K9me3_ESC_DMSO_rep3_R2.fastq.gz
H3K9me3_ESC_dTAG_24h_rep1_R2.fastq.gz
H3K9me3_ESC_dTAG_24h_rep2_R2.fastq.gz
H3K9me3_ESC_dTAG_24h_rep3_R2.fastq.gz
input_ESC_DMSO_rep1_R2.fastq.gz
input_ESC_DMSO_rep2_R2.fastq.gz
input_ESC_DMSO_rep3_R2.fastq.gz
input_ESC_dTAG_24h_rep1_R2.fastq.gz
input_ESC_dTAG_24h_rep2_R2.fastq.gz
input_ESC_dTAG_24h_rep3_R2.fastq.gz
Pol2_ESC_DMSO_rep1_R2.fastq.gz
Pol2_ESC_DMSO_rep2_R2.fastq.gz
Pol2_ESC_dTAG_24h_rep1_R2.fastq.gz
Pol2_ESC_dTAG_24h_rep2_R2.fastq.gz
Med23_ESC_DMSO_rep1_R2.fastq.gz
Med23_ESC_DMSO_rep2_R2.fastq.gz
Med23_ESC_dTAG_24h_rep1_R2.fastq.gz
Med23_ESC_dTAG_24h_rep2_R2.fastq.gz
input_ESC_DMSO_Pol2_R2.fastq.gz
input_ESC_dTAG_24h_Pol2_R2.fastq.gz
input_ESC_DMSO_Med23_R2.fastq.gz
input_ESC_dTAG_24h_Med23_R2.fastq.gz

Genome browser session
(e.g. UCSC)

UCSC track hub: http://ngs.molgen.mpg.de/ngsuploads/dept_meissner/Trim28_Submission.txt

Methodology

Replicates

ChIP-Seq experiments were performed with 2 replicates for RNAPII and MED23 and with 3 replicates for H3K27Ac and H3K9me3. PolyA, Total RNA-seq, and SLAM-TT-seq experiments were performed with 3 biological replicates.

Sequencing depth

READ LENGTH
100 bp, paired-end

READS GENERATED
410687668 H3K27Ac_ESC_DMSO_rep1
110979600 H3K27Ac_ESC_DMSO_rep2
100900068 H3K27Ac_ESC_DMSO_rep3
88916272 H3K27Ac_ESC_dTAG_24h_rep1
95370374 H3K27Ac_ESC_dTAG_24h_rep2
196984348 H3K27Ac_ESC_dTAG_24h_rep3
74250624 H3K9me3_ESC_DMSO_rep1
134359096 H3K9me3_ESC_DMSO_rep2
98332334 H3K9me3_ESC_DMSO_rep3
127067384 H3K9me3_ESC_dTAG_24h_rep1
111877386 H3K9me3_ESC_dTAG_24h_rep2
142408192 H3K9me3_ESC_dTAG_24h_rep3
52207990 input_ESC_DMSO_rep1
54628918 input_ESC_DMSO_rep2
48538948 input_ESC_DMSO_rep3
21402648 input_ESC_dTAG_24h_rep1
50348032 input_ESC_dTAG_24h_rep2
59192212 input_ESC_dTAG_24h_rep3
52504118 Pol2_ESC_DMSO_rep1
60827162 Pol2_ESC_DMSO_rep2
52535240 Pol2_ESC_dTAG_24h_rep1
23526768 Pol2_ESC_dTAG_24h_rep2
61338540 Med23_ESC_DMSO_rep1
116714578 Med23_ESC_DMSO_rep2

86450084 Med23_ESC_dTAG_24h_rep1
72728384 Med23_ESC_dTAG_24h_rep2
13488990 input_ESC_DMSO_Pol2
8964686 input_ESC_dTAG_24h_Pol2
22051410 input_ESC_DMSO_Med23
10273856 input_ESC_dTAG_24h_Med23

READS UNIQUELY ALIGNED MOUSE (mm10)
344164541 H3K27Ac_ESC_DMSO_rep1
96585004 H3K27Ac_ESC_DMSO_rep2
79503843 H3K27Ac_ESC_DMSO_rep3
72703368 H3K27Ac_ESC_dTAG_24h_rep1
79715499 H3K27Ac_ESC_dTAG_24h_rep2
171498224 H3K27Ac_ESC_dTAG_24h_rep3
57983745 H3K9me3_ESC_DMSO_rep1
111284700 H3K9me3_ESC_DMSO_rep2
76982969 H3K9me3_ESC_DMSO_rep3
102970188 H3K9me3_ESC_dTAG_24h_rep1
89778418 H3K9me3_ESC_dTAG_24h_rep2
114868841 H3K9me3_ESC_dTAG_24h_rep3
45427465 input_ESC_DMSO_rep1
46429874 input_ESC_DMSO_rep2
38190218 input_ESC_DMSO_rep3
15313320 input_ESC_dTAG_24h_rep1
15313320 input_ESC_dTAG_24h_rep2
44597123 input_ESC_dTAG_24h_rep3
35279218 Pol2_ESC_DMSO_rep1
39964121 Pol2_ESC_DMSO_rep2
35342996 Pol2_ESC_dTAG_24h_rep1
14297274 Pol2_ESC_dTAG_24h_rep2
49903588 Med23_ESC_DMSO_rep1
107669156 Med23_ESC_DMSO_rep2
73871774 Med23_ESC_dTAG_24h_rep1
65622785 Med23_ESC_dTAG_24h_rep2
5610583 input_ESC_DMSO_Pol2
2358339 input_ESC_dTAG_24h_Pol2
19483039 input_ESC_DMSO_Med23
5433101 input_ESC_dTAG_24h_Med23

READS UNIQUELY ALIGNED DROSOPHILA (dm6)
44597123 H3K27Ac_ESC_DMSO_rep1
11659503 H3K27Ac_ESC_DMSO_rep2
11051087 H3K27Ac_ESC_DMSO_rep3
9505642 H3K27Ac_ESC_dTAG_24h_rep1
10509582 H3K27Ac_ESC_dTAG_24h_rep2
22429630 H3K27Ac_ESC_dTAG_24h_rep3
10520245 H3K9me3_ESC_DMSO_rep1
17902550 H3K9me3_ESC_DMSO_rep2
13217409 H3K9me3_ESC_DMSO_rep3
20398860 H3K9me3_ESC_dTAG_24h_rep1
20398860 H3K9me3_ESC_dTAG_24h_rep2
21341601 H3K9me3_ESC_dTAG_24h_rep3
11451871 input_ESC_DMSO_rep1
11024536 input_ESC_DMSO_rep2
9499176 input_ESC_DMSO_rep3
4831231 input_ESC_dTAG_24h_rep1
9254723 input_ESC_dTAG_24h_rep2
13725562 input_ESC_dTAG_24h_rep3
16837010 Pol2_ESC_DMSO_rep1
20002855 Pol2_ESC_DMSO_rep2
18386451 Pol2_ESC_dTAG_24h_rep1
7731020 Pol2_ESC_dTAG_24h_rep2
1197237 input_ESC_DMSO_Pol2
1197237 input_ESC_dTAG_24h_Pol2

Antibodies

RNAPII (8WG16; Biolegend)

Spike-in antibody (61686, Active Motif)

MED23 antibody (A300-425A; Bethyl Laboratories)

H3K27Ac (ab4729; Abcam)

H3K9me3 (ab8898; Abcam)

Peak calling parameters

MED23
Raw reads of treatment and input samples were subjected to adapter and quality trimming with cutadapt (version 2.4; parameters: --nextseq-trim 20 --overlap 5 --minimum-length 25 --adapter AGATCGGAAGAGC -A AGATCGGAAGAGC). Reads were aligned to the mouse genome (mm10) using bwa with the 'mem' command (version 0.7.17, default parameters). A sorted BAM file was obtained and indexed using samtools with the 'sort' and 'index' commands (version 1.10). Duplicate reads were identified and removed using gatk (version 4.1.4.1) with the 'MarkDuplicates' command and default parameters. Technical replicates of treatment and input samples were merged respectively using samtools 'merge'.
Peaks were called with reads aligning to the mouse genome using MACS2 'callpeak' (version 2.1.2; parameters --bdg --SPMR) using the input samples as control samples. Genome-wide coverage tracks for single and merged replicates normalized by library size were computed using bamCoverage (version: 3.4.3; parameters: --normalizeUsing CPM --extendReads).

ALL OTHER
Raw reads of treatment and input samples were subjected to adapter and quality trimming with cutadapt (version 2.4; parameters: --nextseq-trim 20 --overlap 5 --minimum-length 25 --adapter AGATCGGAAGAGC -A AGATCGGAAGAGC). Reads were aligned separately to the mouse genome (mm10) and to the fly genome (D. Melanogaster, dm6) using bwa with the 'mem' command (version 0.7.17, default parameters). A sorted BAM file was obtained and indexed using samtools with the 'sort' and 'index' commands (version 1.10). Duplicate reads were identified and removed using gatk (version 4.1.4.1) with the 'MarkDuplicates' command and default parameters. Technical replicates of treatment and input samples were merged respectively using samtools 'merge'.
Peaks were called with reads aligning to the mouse genome only using MACS2 'callpeak' (version 2.1.2; parameters --bdg --SPMR) using the input samples as control samples. For H3K9me3 only, the '--broad' option was used. Genome-wide coverage tracks for single and merged replicates normalized by library size were computed using bamCoverage (version: 3.4.3; parameters: --normalizeUsing CPM --extendReads) and in addition normalized by the spike-in factor obtained from the reads aligning to the drosophila genome.

Data quality

Quality of raw reads was assessed using FastQC. Reads were trimmed using cutadapt in order to remove low-quality bases and adapter content.

FRACTION ALIGNED TO MOUSE (mm10) - The fraction might deviate from the numbers noted above due to reads being excluded after the trimming process.

0.93 H3K27Ac_ESC_DMSO_rep1
0.93 H3K27Ac_ESC_DMSO_rep2
0.93 H3K27Ac_ESC_DMSO_rep3
0.93 H3K27Ac_ESC_dTAG_24h_rep1
0.93 H3K27Ac_ESC_dTAG_24h_rep2
0.94 H3K27Ac_ESC_dTAG_24h_rep3
0.90 H3K9me3_ESC_DMSO_rep1
0.91 H3K9me3_ESC_DMSO_rep2
0.91 H3K9me3_ESC_DMSO_rep3
0.89 H3K9me3_ESC_dTAG_24h_rep1
0.89 H3K9me3_ESC_dTAG_24h_rep2
0.90 H3K9me3_ESC_dTAG_24h_rep3
0.89 input_ESC_DMSO_rep1
0.89 input_ESC_DMSO_rep2
0.89 input_ESC_DMSO_rep3
0.91 input_ESC_dTAG_24h_rep1
0.90 input_ESC_dTAG_24h_rep2
0.84 input_ESC_dTAG_24h_rep3
0.75 Pol2_ESC_DMSO_rep1
0.73 Pol2_ESC_DMSO_rep2
0.73 Pol2_ESC_dTAG_24h_rep1
0.71 Pol2_ESC_dTAG_24h_rep2
0.99 Med23_ESC_DMSO_rep1
0.99 Med23_ESC_DMSO_rep2
0.99 Med23_ESC_dTAG_24h_rep1
0.99 Med23_ESC_dTAG_24h_rep2
0.91 input_ESC_DMSO_Pol2
0.89 input_ESC_dTAG_24h_Pol2
0.99 input_ESC_DMSO_Med23
0.98 input_ESC_dTAG_24h_Med23

Software

cutadapt
bwa mem
samtools
gatk
MACS2
bamCoverage

Flow Cytometry

Plots

Confirm that:

☒ The axis labels state the marker and fluorochrome used (e.g. CD4-FITC).

☒ The axis scales are clearly visible. Include numbers along axes only for bottom left plot of group (a 'group' is an analysis of identical markers).

☐ All plots are contour plots with outliers or pseudocolor plots.

☒ A numerical value for number of cells or percentage (with statistics) is provided.

Methodology

Sample preparation	Cells were blocked with 10% FCS for 15 minutes followed by incubation with primary antibody for one hour at 37 degree C. This was followed by two washes at room temperature for 10 minutes each and measured with the flow cytometer.
Instrument	BD FACS Celesta, BD FACS Aria II
Software	FACS Diva (BD Biosciences) for collection and FlowJo (v1.07) for analysis
Cell population abundance	For each sample, a population of at least 10,000-30,000 cells was obtained, and the overall cell population was calculated using forward and side-scatter patterns. The abundance of cells in a population is represented as the normalized mode.
Gating strategy	Gating for negative and positive population was determined with untreated or isotype controls.

☐ Tick this box to confirm that a figure exemplifying the gating strategy is provided in the Supplementary Information.
