# Supplementary figures and images for: Hijacking of transcriptional condensates by endogenous retroviruses
Source: Nat Genet. 2022 Jul 21;54(8):1238–47. doi: 10.1038/s41588-022-01132-w (PMC9355880; doi:10.1038/s41588-022-01132-w)

Supplementary Data 1

Uncropped data of gel images from Supplementary Figure 2b

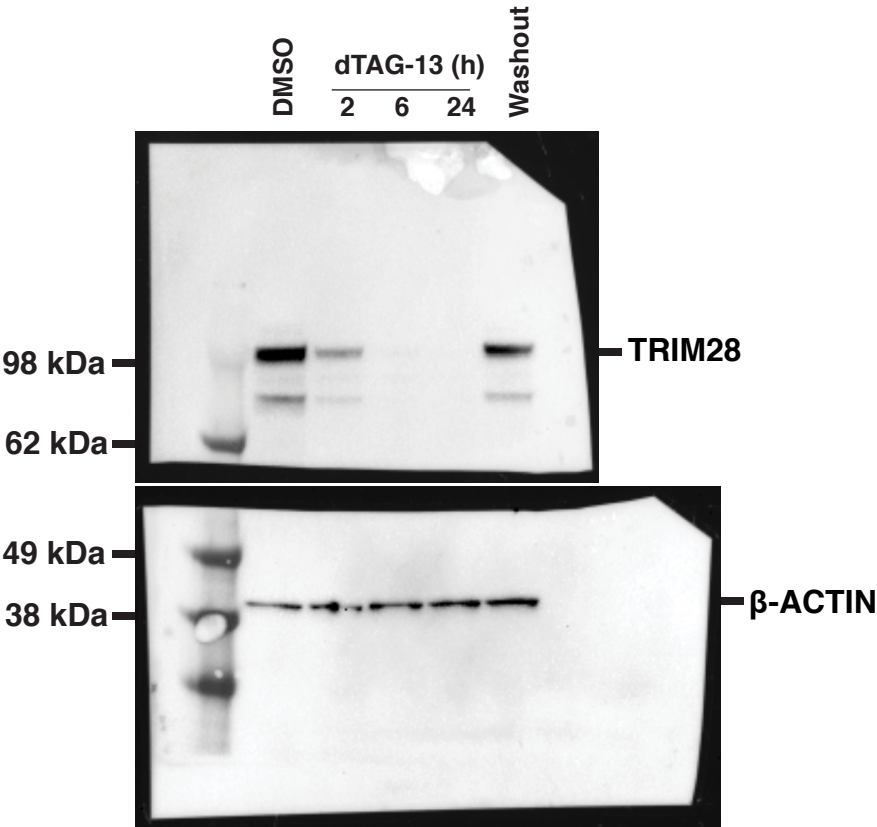

Supplement: Supplementary file 5 — Uncropped gel images for Supplementary Fig. 2b [file 41588_2022_1132_MOESM5_ESM.pdf]

Supplementary Data 2

Uncropped data of gel images from Supplementary Figure 2m

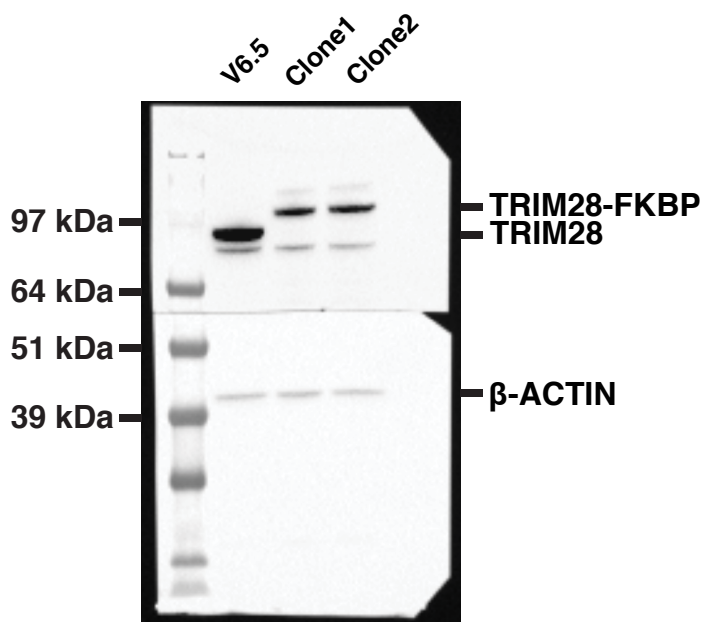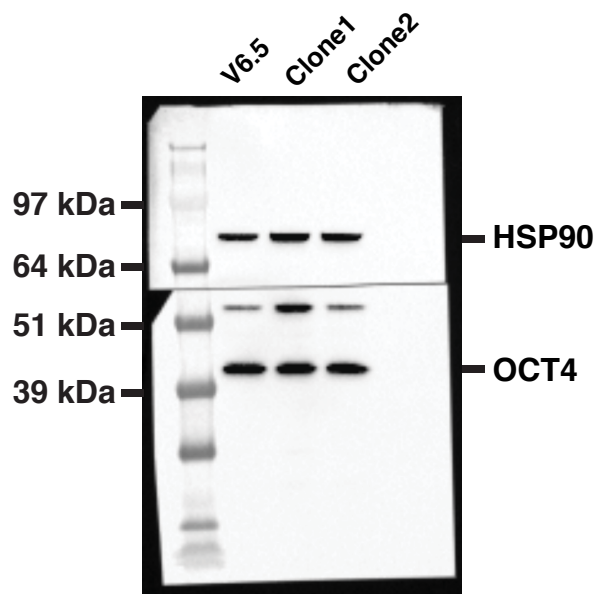

Supplement: Supplementary file 6 — Uncropped gel images for Supplementary Fig. 2m. [file 41588_2022_1132_MOESM6_ESM.pdf]

Supplementary Data 3

Uncropped gel image from Supplementary Figure 6b

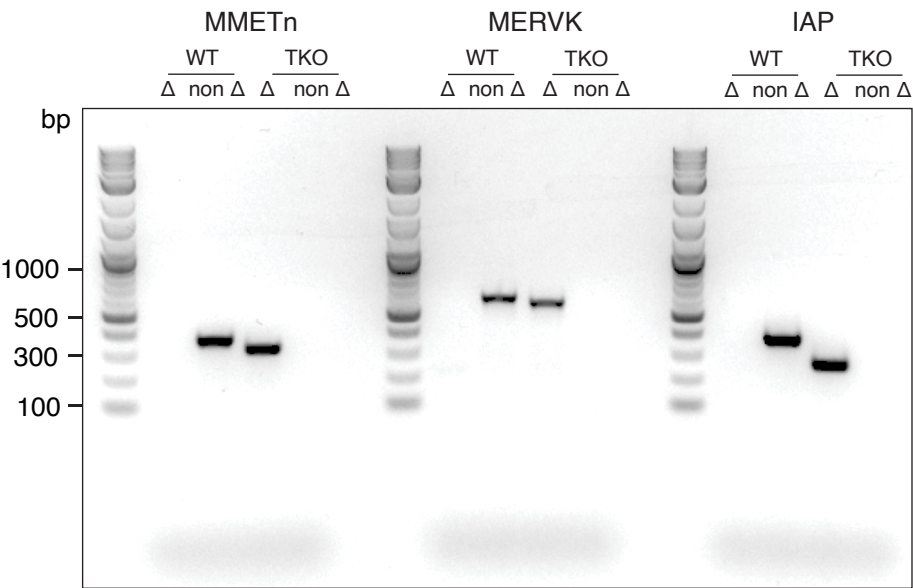

Supplement: Supplementary file 7 — Uncropped gel images for Supplementary Fig. 6b. [file 41588_2022_1132_MOESM7_ESM.pdf]

**Uncropped data of gel images from Figure 1d . Blots are shown in the same order as in the main figure.**

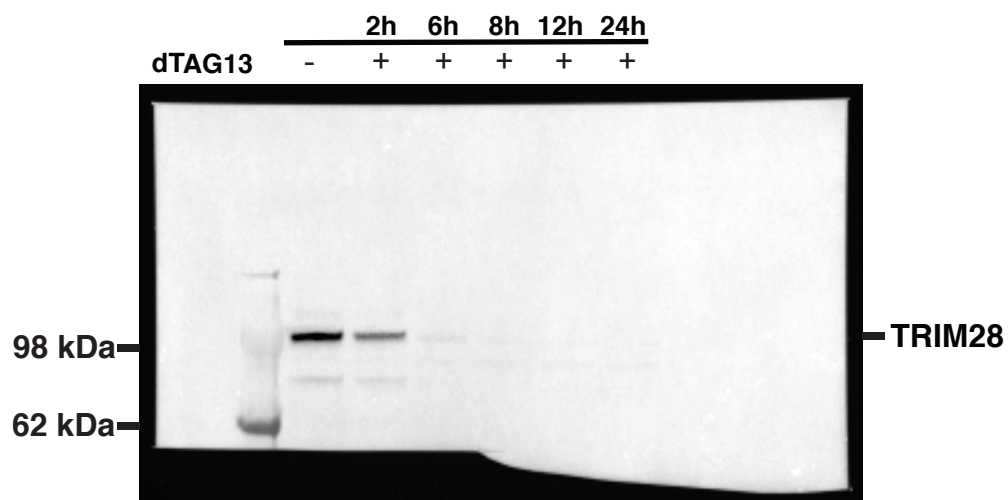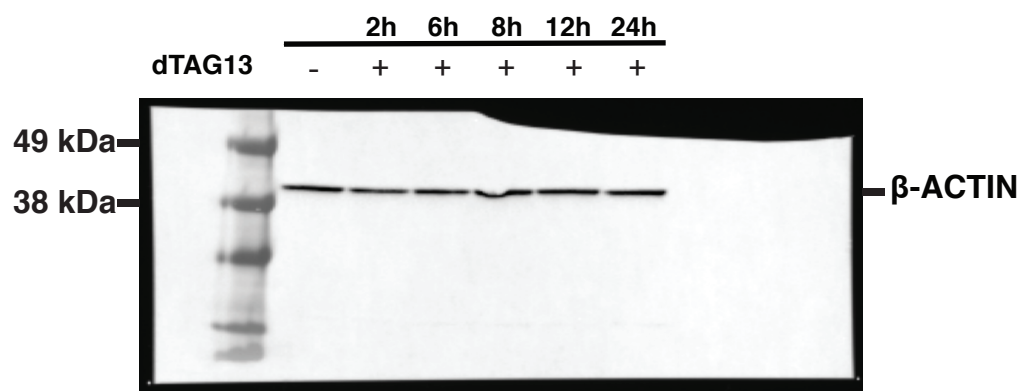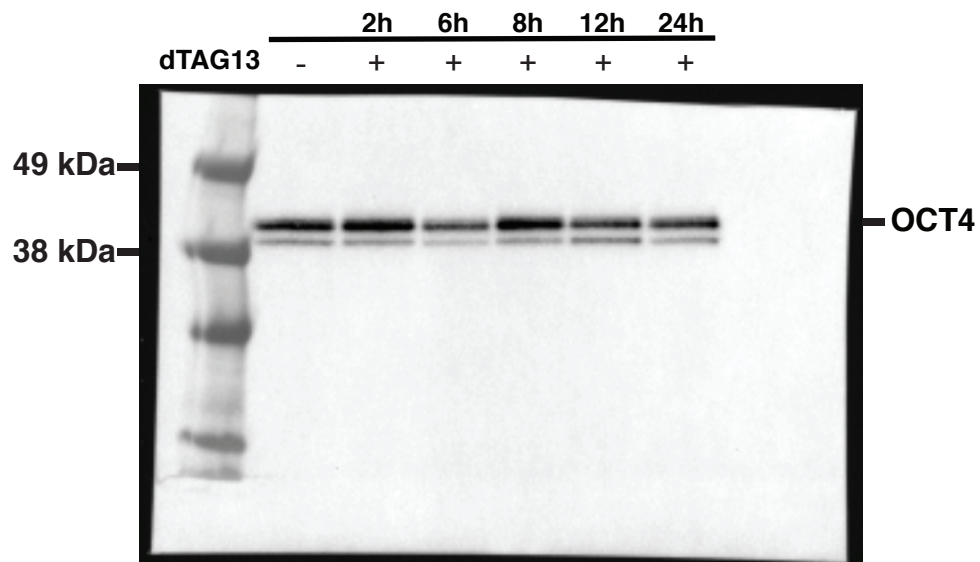

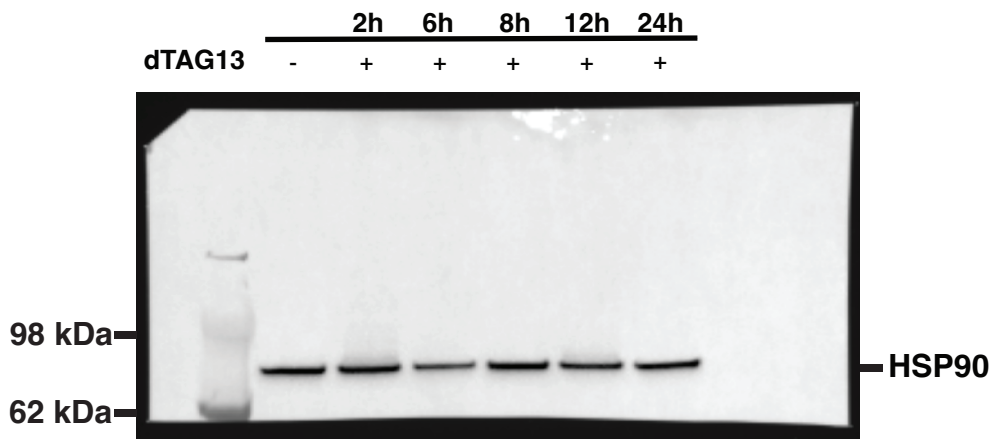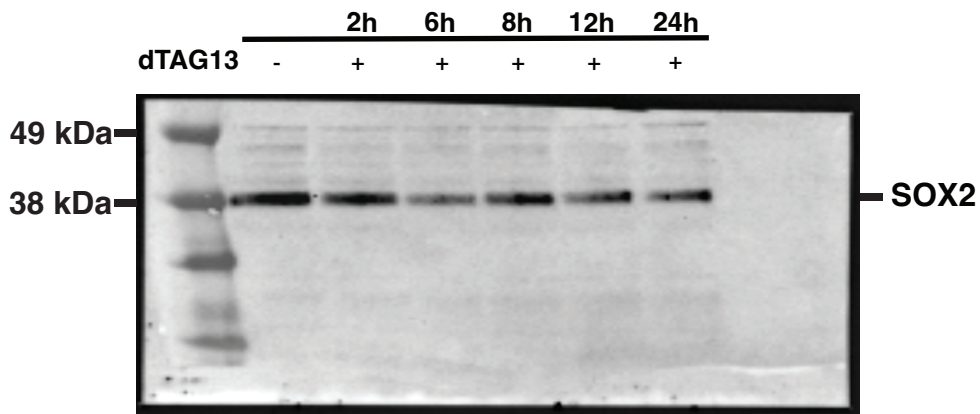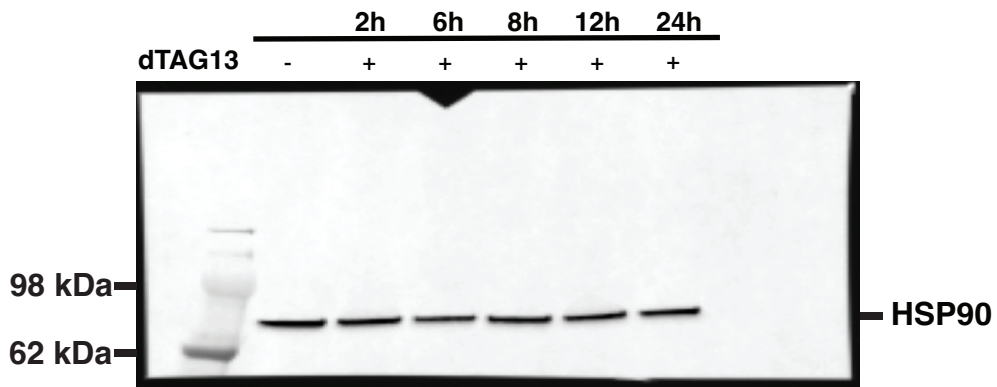

Supplement: Source Data Fig. 1 — Uncropped blot images for Fig. 1d. [file 41588_2022_1132_MOESM8_ESM.pdf]

Uncropped data of gel images from Figure 3b.

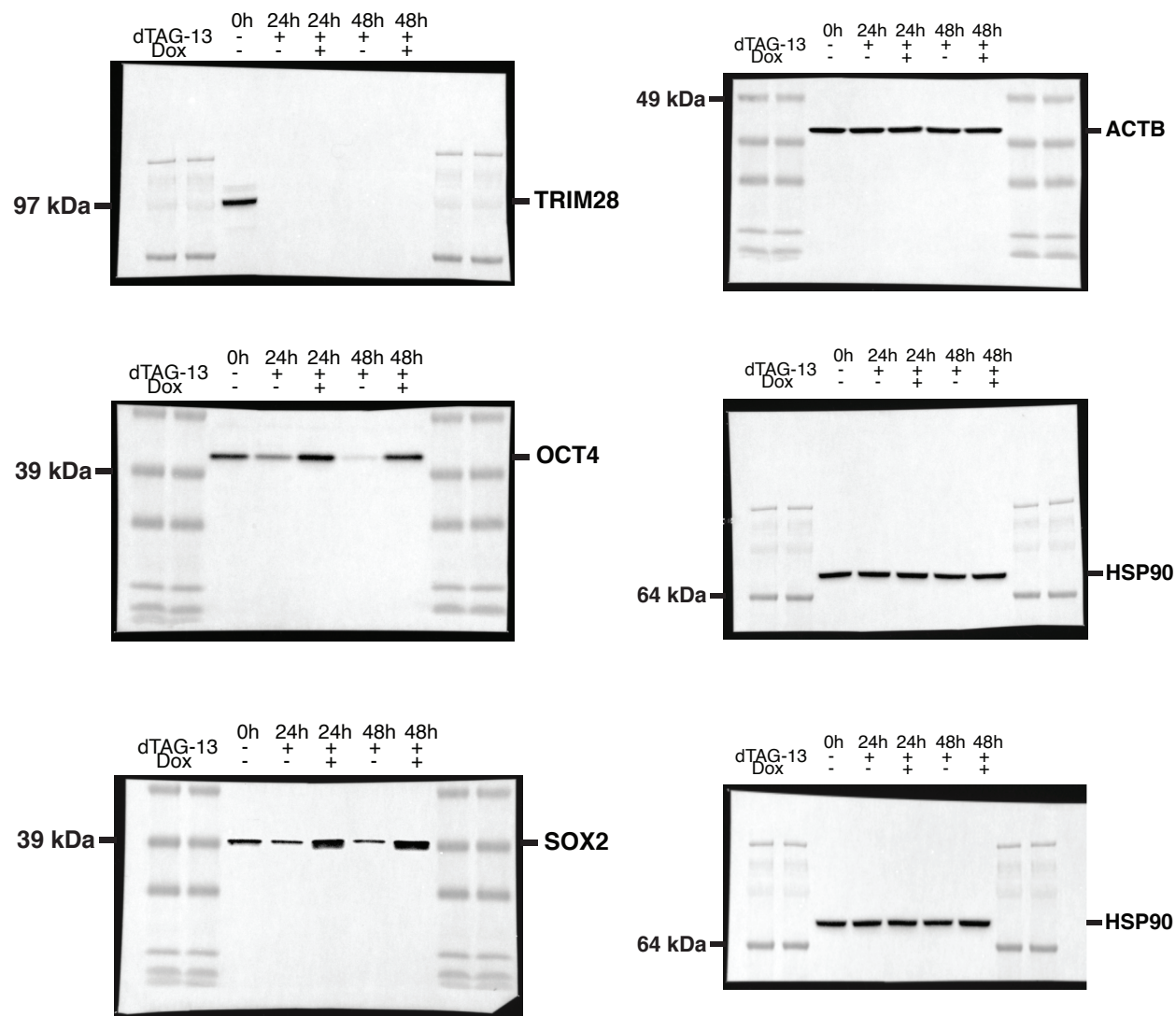

Supplement: Source Data Fig. 3 — Uncropped blot images for Fig. 3b. [file 41588_2022_1132_MOESM9_ESM.pdf]

Uncropped data of gel image from Extended Data Figure 2f

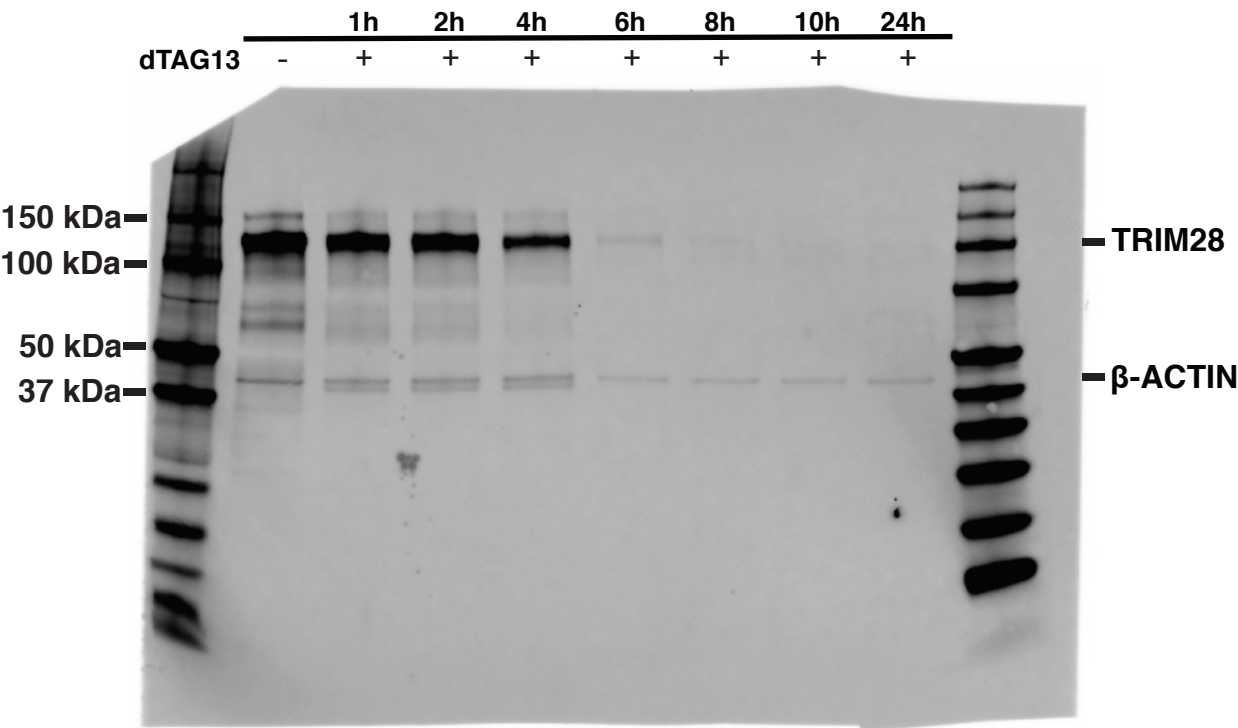

Supplement: Source Data Extended Data Fig. 2 — Uncropped blot images for Extended Data Fig. 2f. [file 41588_2022_1132_MOESM10_ESM.pdf]

Uncropped data of gel images from Extended Data Figure 9a.

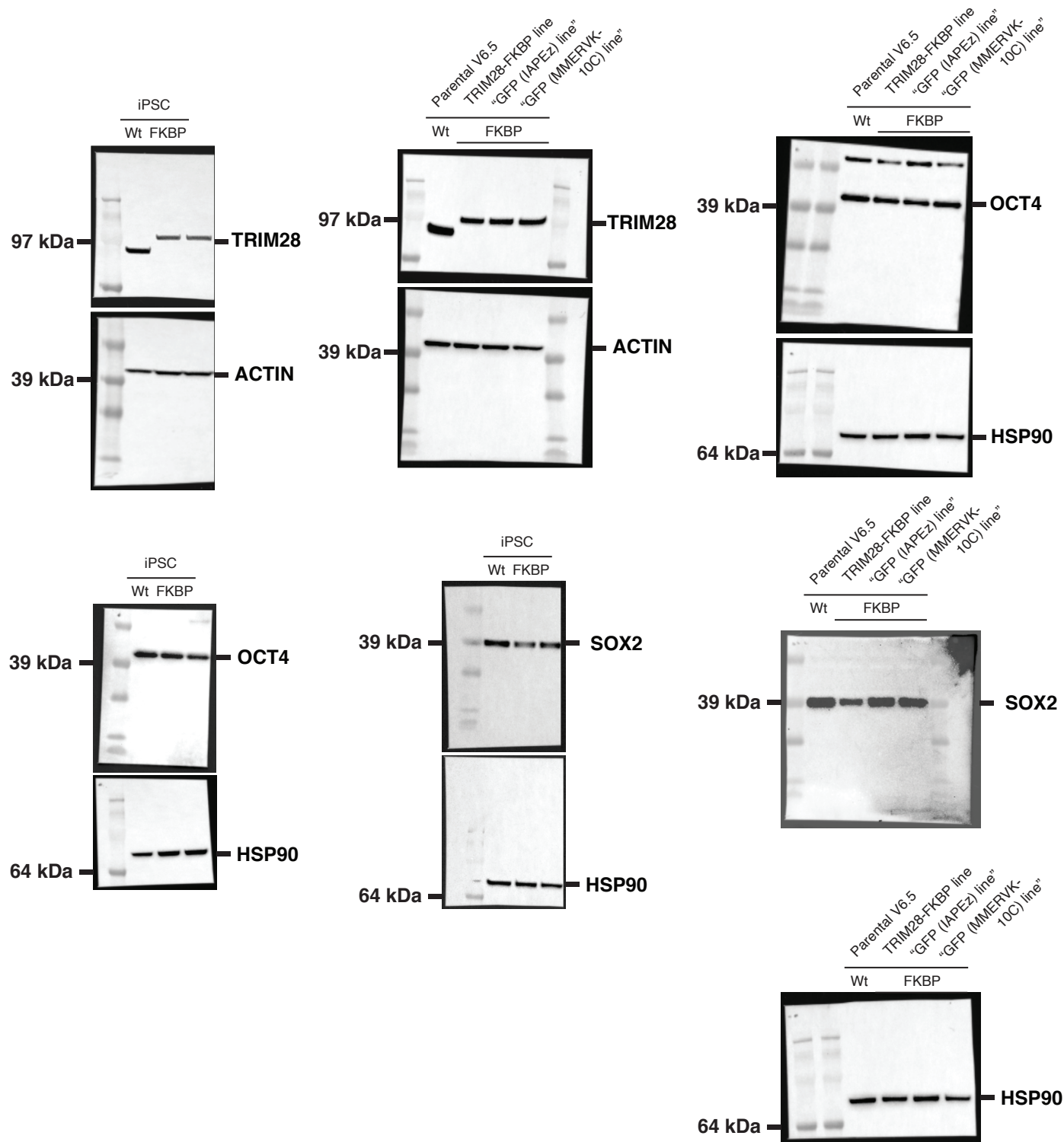

Supplement: Source Data Extended Data Fig. 9 — Uncropped blot images for Extended Data Fig. 9a. [file 41588_2022_1132_MOESM12_ESM.pdf]
