# Supplementary material for: Hijacking of transcriptional condensates by endogenous retroviruses
Source: Nat Genet. 2022 Jul 21;54(8):1238–47. doi: 10.1038/s41588-022-01132-w (PMC9355880; doi:10.1038/s41588-022-01132-w)

Uncropped data of gel images from Extended Data Figure 5a.

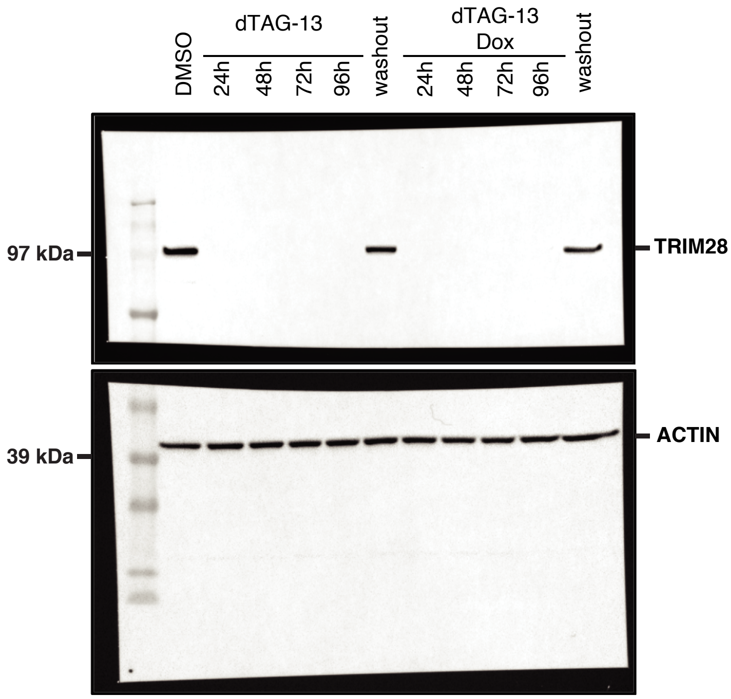

Uncropped data of gel images from Extended Data Figure 5b.

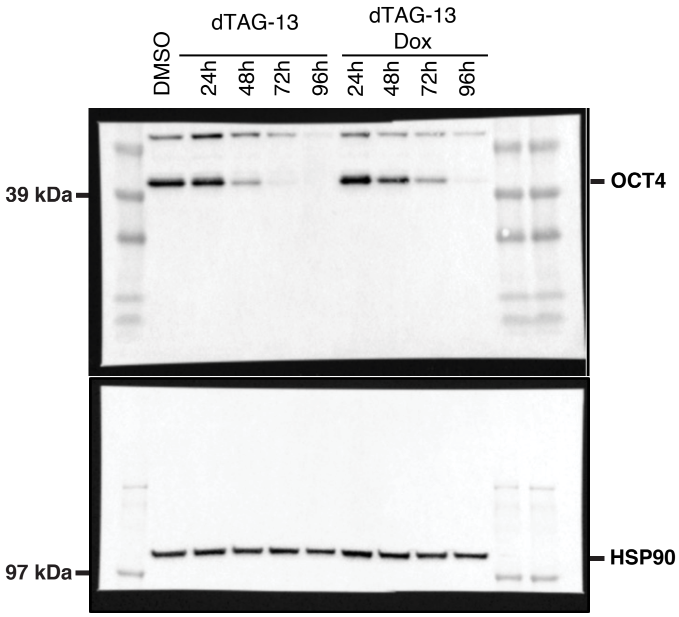

Supplement: Source Data Extended Data Fig. 5 — Uncropped blot images for Extended Data Fig. 5a,b. [file 41588_2022_1132_MOESM11_ESM.pdf]
